# Capsicum Leaves under Stress: Using Multi-Omics Analysis to Detect Abiotic Stress Network of Secondary Metabolism in Two Species

**DOI:** 10.3390/antiox11040671

**Published:** 2022-03-30

**Authors:** Julia Jessica Reimer, Basel Shaaban, Noud Drummen, Sruthy Sanjeev Ambady, Franziska Genzel, Gernot Poschet, Anika Wiese-Klinkenberg, Björn Usadel, Alexandra Wormit

**Affiliations:** 1 Institute for Biology I, RWTH Aachen University, Worringer Weg 3, 52074 Aachen, Germany; bshaaban@bio1.rwth-aachen.de (B.S.); nouddrummen@gmail.com (N.D.); sruthy.sanjeev.ambady@rwth-aachen.de (S.S.A.); b.usadel@fz-juelich.de (B.U.); awormit@bio1.rwth-aachen.de (A.W.); 2 Faculty of Technology, Molecular Biosciences, University of Applied Science Emden/Leer, Constantiaplatz 4, 26723 Emden, Germany; 3 Institute of Bio- and Geosciences, IBG-2: Plant Sciences, Forschungszentrum Jülich, 52425 Jülich, Germany; f.genzel@fz-juelich.de (F.G.); a.wiese@fz-juelich.de (A.W.-K.); 4 Institute of Bio- and Geosciences, IBG-4: Bioinformatics, Forschungszentrum Jülich, 52425 Jülich, Germany; 5 Centre for Organismal Studies, University of Heidelberg, Im Neuenheimer Feld 360, 69120 Heidelberg, Germany; gernot.poschet@cos.uni-heidelberg.de; 6 Chair of Biological Data Science, Heinrich-Heine-University, 40225 Düsseldorf, Germany

**Keywords:** bell pepper, capsicum, flavonoid, transcriptome, metabolome, abiotic stress

## Abstract

The plant kingdom contains an enormous diversity of bioactive compounds which regulate plant growth and defends against biotic and abiotic stress. Some of these compounds, like flavonoids, have properties which are health supporting and relevant for industrial use. Many of these valuable compounds are synthesized in various pepper (*Capsicum* sp.) tissues. Further, a huge amount of biomass residual remains from pepper production after harvest, which provides an important opportunity to extract these metabolites and optimize the utilization of crops. Moreover, abiotic stresses induce the synthesis of such metabolites as a defense mechanism. Two different *Capsicum* species were therefore exposed to chilling temperature (24/18 ℃ vs. 18/12 ℃), to salinity (200 mM NaCl), or a combination thereof for 1, 7 and 14 days to investigate the effect of these stresses on the metabolome and transcriptome profiles of their leaves. Both profiles in both species responded to all stresses with an increase over time. All stresses resulted in repression of photosynthesis genes. Stress involving chilling temperature induced secondary metabolism whereas stresses involving salt repressed cell wall modification and solute transport. The metabolome analysis annotated putatively many health stimulating flavonoids (apigetrin, rutin, kaempferol, luteolin and quercetin) in the *Capsicum* biomass residuals, which were induced in response to salinity, chilling temperature or a combination thereof, and supported by related structural genes of the secondary metabolism in the network analysis.

## 1. Introduction

The genus *Capsicum* belongs to the Solanaceae family, and comprises five major domesticated species with a broad diversity in fruit morphology and flavour—*Capsicum annuum* L., *C. baccatum* L., *C. chinense* Jacq., *C. frutescens* L. and *C. pubescens* Ruiz and Pav.—and about 37 wild species which have small, berry-like fruits [1]. *Capsicum* originated from Central/South America and Mexico. It was domesticated and later spread around the globe to Europe, Asia and Africa [2]. As a tropical and subtropical annual crop, it requires a warm (18–30 ℃) and humid climate for optimal growth and fruit production [3], but can also be cultivated in temperate climates in greenhouses [4].

*Capsicum annuum* is globally the most cultivated species and the worldwide production of chilis and peppers has increased from about 7 million tonnes to more than 40 million tonnes in the last 50 years, due to their role as both spice and vegetable [5]. *Capsicum* fruits have a high nutritional content and are an important and rich source of bioactive compounds (so-called plant secondary metabolites, short PSM) such as phenols, carotenoids and flavonoids, as well as capsaicinoids which determine the variable levels of pungency [6]. PSM in general have beneficial properties, such as antioxidant [7,8,9], anti-inflammatory [10], antimicrobial [11,12] or even anti-Alzheimer disease [13] and anti-cancer qualities [14,15], which is of interest for the cosmetic, food and pharmaceutic industry. Few studies also detected PSM contents in the leaves of *Capsicum* species (for review, see Carvalho Lemos et al. [16]). Considering the aforementioned production volume of chilis and peppers, the residual leaf biomass could be utilized as a valuable source for extraction of bioactive compounds for industrial applications [17] as was proposed for tomato [18,19,20].

Extreme environmental conditions negatively affect crop growth and development [21,22,23,24]. Therefore, understanding the mechanisms of plant responses to environmental stresses will contribute to improving crop stress resistance and increasing crop yield and quality. High salinity adversely impacts plants by inducing osmotic and ionic stress, caused by water deficiency and consequently reduced absorption of nutrients [24,25,26]. This leads to oxidative stress causing damage to cellular membranes, macromolecules like proteins and nucleic acids, leading to inhibition of photosynthesis, metabolic dysfunction and repression of cell division and expansion [27]. To avoid oxidative stress damage, plants activate antioxidant enzymes like superoxide dismutase (SOD), peroxidase (POD), catalase (CAT) and accumulate non-enzymatic antioxidant compounds, including PSM like flavonoids, phenolic acids, carotenoids or alkaloids [28,29]. Chilling and cold temperature may also cause water deficiency due to poor root hydraulic conductance and diminished root activity [30], resulting in oxidative stress [28]. Consequently, plants activate the antioxidant defense system in response to cold stress [28]. Bell pepper plants have been shown to accumulate flavonoids, phenolic acids and carotenoids in response to chilling temperature treatment [31]. The induced increase in carotenoid levels by chilling temperature was found to be regulated by the transcription factor gene *CaATHB-12* in *Capsicum* fruits [32]. Furthermore, plants can be exposed to different stresses at the same time under field conditions, which is even worse for crop production rather than an individual stress condition [33]. Combination of stresses results in common and different physiological and molecular adaptations compared to single stresses [34,35]. Therefore, an understanding of the unique responses to combined stress treatments is necessary for developing stress tolerant crop plants [34].

Conventional breeding has allowed the development of crop cultivars with greater tolerance to stress, for example to cold stress, drought or salinity [36,37,38]. Wild relatives of crop species represent an important resource for stress adaptation traits as many of them are adapted to extreme climates, adverse soil types, and important pests and diseases [39]. It has been shown that non-domesticated species often contain higher levels of polyphenolics than cultivated varieties, suggesting a better antioxidant defense system [40,41]. However, knowledge gaps with regard to wild genetic resources are hindering their potential use in plant breeding. In accordance, the genetic diversity of wild relatives of domesticated *Capsicum* has not been well studied [42] and their tolerance to abiotic stress has not been thoroughly investigated [1]. *Capsicum chinense* is a species originating from South America that is a putative progenitor or a close relative of the domesticated *C. annuum* taxa. Some taxonomists consider them to be part of the *C. annuum* complex [43,44]. It is occasionally cultivated in home gardens and perennial in warm climates, but will not survive the winter in chilling climates.

Multi-omics approaches can utilize data from different omics sources and reveal the correlation between them [45]. Modern technologies like next-generation sequencing and different untargeted mass spectrometry approaches provide high-throughput genomics, transcriptomics, proteomics and metabolomics data [46]. These huge data sets can be very useful to address and investigate different biological issues, such as identification of key changes to metabolic pathways. However, in plants the interpretation of omics data is challenging due to the huge number of unknown mass profiles and non-annotated genes [47]. The utilization of multi-omics approaches in non-model species can be advantageous to reveal the correlation between different omics profiles and identification of genes, proteins and metabolites of interest [48,49,50]. For example, by performing multivariate discriminant analyses method (DIABLO, Data Integration Analysis for Biomarker discovery using a Latent component method for Omics studies) [51], proteomics and metabolomics data were used to identify correlated variables to heat stress in avocado [50].

In this study, we investigated the adaptation responses of cultivated bell pepper *C. annuum* to the single stresses of salinity and chilling temperatures as well as the combinatorial stress and compared those to the relative *C. chinense*. More precisely, a cultivated bell pepper (Mazurka) and a chili (CAP 1035) (both as young plants) were exposed to chilling temperatures (day 18 ℃, night 12 ℃), to salinity (200 mM NaCl), or a combination thereof. Leaf samples were harvested after 1, 7 and 14 days and analyzed regarding their metabolome profiles using untargeted metabolomics approaches via LC-MS, and transcriptome profiles by using high-throughput sequencing. Then, different multivariate analyses were performed on the transcriptome and metabolome profiles, independently, to study the effect of different species, treatments and harvest time points on each profile. Finally, using DIABLO, transcriptome and metabolome profiles were analyzed together to identify correlated key omics variables for abiotic stress in each species in particular for the secondary metabolism.

## 2. Materials and Methods

### 2.1. Plant Cultivation

*Capsicum annuum* L. var. *annuum* cv. Mazurka (Rijk Zwaan Welver GmbH, Germany) and *Capsicum chinense* Jacq. cv. CAP 1035 (Genetics Resource Center IPK Gatersleben, Germany) were grown from seeds in environmental chambers (Hühren Kälte-Klima-Elektrotechnik, Erkelenz, Germany), equipped with metal halide lamps (Philips, Hamburg, Germany), following the protocol described elsewhere [52], under controlled conditions of 24/18 ℃ during day and night, a relative humidity of 55% and 300 µmol m^−2^ s^−1^ photons light intensity for 10 h per day.

In brief, plants were sowed in rock wool plugs (2 × 2 × 4 cm; Grodan, Roermond, The Netherlands), that were four times prewashed with deionized water. Seeds were placed around 0.5 cm below the surface of the rock wool plugs. The plugs were watered with 1/4 Hoagland solution for 14 days, followed by half-strength Hoagland solution for 14 days. A total of 54 seedlings per species, which had already developed the first real leaf, were transferred to rock wool blocks (7.5 × 7.5 × 6.5 cm) (Grodan, Roermond, The Netherlands), that were 3 times prewashed with deionized water. Afterwards, the seedlings were fertilized with full-strength Hoagland solution (5 mM KNO_3_, 5 mM Ca(NO_3_)_2_, 2 mM MgSO_4_, 1 mM KH_2_PO_4_, 90 µM FeEDTA, plus micronutrients).

### 2.2. Abiotic Stress Treatments and Harvesting

**Figure A1:**
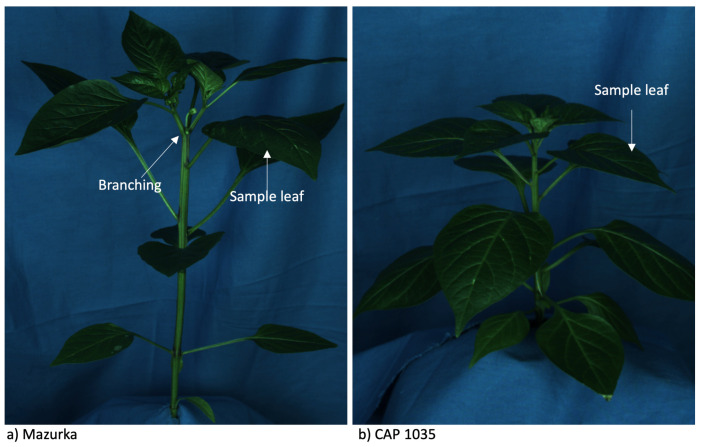
Position of the harvested leaf in Mazurka (**a**) and CAP 1035 (**b**). Leaves beneath the first branching (seen from the tip of the plant) were harvested in both species. Indicated are the leaf and for Mazurka as well the branching point, as visible in the photo.

Eight weeks after germination, plants were stressed by chilling temperatures, watering with Hoagland solution containing additional 200 mM NaCl or a combination of both. Chilling temperatures were achieved by transferring plants to an identical environmental walk-in chamber set to 18/12 ℃ (day/night). All plants in chambers were randomized once a week. The stress treatments were performed in two independent experiments. One day (d1), 7 days (d7) and 14 days (d14) after induction of stress treatments, the leaf beneath first branching was sampled (see also Figure A1), immediately frozen in liquid nitrogen and stored at −80 ℃.

A total number of 48 pepper plants per species were harvested. At d1, d7 and d14 after the beginning of the stress treatment four plants per stress and four control plants were harvested each.

Leaf material was ground to fine powder in liquid nitrogen with an MM400 mixer mill in a 25 mL grinding beaker with two 15 mm diameter grinding balls (Retsch, Haan, Germany) for 30 s at a frequency of 30 s^−1^. Afterwards the samples were stored at −80 ℃.

### 2.3. Total RNA Extraction and DNase-Digestion

RNA was extracted using a modified column based kit from QIAGEN GmbH (Plant RNeasy, QIAGEN GmbH, Hilden, Germany). Briefly, 50–100 mg cooled sample powder was mixed with 1 mL Trizol using a Retsch machine (150 s, 30 Hz, room temperature), and further incubated for 150 s at room temperature. Then, 200 µL chloroform were added and shortly mixed vigorously before spinning the mixture at 4 ℃ and full speed for 15 min in a table centrifuge. The aqueous phase was mixed 1:1 (v:v) with 100% ethanol and loaded on the column. Afterwards, the manufacturer’s instruction was followed. The received total RNA was eluted in 30–40 µL RNase-free water. Total RNA was stored at −80 ℃ for long term storage and −20 ℃ for short term storage. The concentration and quality of the total RNA was determined by a Nanodrop measurement (Nanodrop 2000c, Thermo Fischer Scientific GmbH, Schwerte, Germany) and a Bioanalyzer analysis (Agilent, Santa Clara, CA, USA). The achieved RNA integrity numbers (RIN) of our samples were in the range of 6.8 ± 0.4.

To remove residual genomic DNA in the total RNA samples, a DNase digest was performed using Baseline-ZERO™ DNase (Epicentre, Madison, WI, USA). 3.4 µL of the reaction buffer was added to 30 µL total RNA, in addition to 1 µL DNase (1 MBU). The digest was performed at 37 ℃ for 30 min. The DNase was inactivated by adding 4 µL stop buffer and an incubation at 70 ℃ for 10 min. Afterwards, total RNA was precipitated at −20 ℃ for 2 days using 100 µL ice-cold isopropanol. The sample was washed twice using 70% ethanol, air dried for 10 min at room temperature and resuspended in 30–40 µL RNase-free water.

The RNA was used for RNASeq and further quantified using cDNA synthesis and a quantitative real time PCR approach.

### 2.4. RNASeq Data Handling and Analysis

mRNA was enriched from total RNA samples, and subsequently analyzed by the commercial provider Novogene using an Illumina-platform (NovaSeq 6000) to sequence 2 × 150 bp paired-end reads.

Raw reads were trimmed using Trimmomatic [53]. Reads were aligned to the genome of *C. annuum* cv. Zunla-1 using hisat 2 (version 2.1.0) and Salmon [54,55]. The reference genome for *C. annuum* cv. Zunla-1 was published by Qin et al. [56].

An artificial transcriptome was built using default settings of StringTie [57,58] with trimmed reads of all analyzed conditions, to investigate how similar the two species behave in a principal component analysis (PCA) and further analysis. In addition, the artificial transcriptome was expanded using trimmed reads from a publication of Kang et al. [59] to check for similarities between the two independent data sets.

Further, read abundancy was calculated by using Salmon. Data analysis was performed using R 3.5.2 [60]. Read abundancies were analyzed using R-packages limma [61], edgeR [62,63], and tximport [64]. The full list of count per million (cpm) and expression ratios can be found in supplementary Table S1.

Overrepresentation analysis was carried out with PageMan [65]. Weighted cluster analysis (WGCNA) was performed using the R-packageWGCNA [66] (with default settings, softpower = 9, minModuleSize = 30, and mergeCutHeight = 0.25) with the following traits: chilling temperature and salt treatment. During the analysis with WGCNA, genes with a low coefficient of variation among all sample types were discarded and the remaining genes were used for the analysis.

### 2.5. cDNA Synthesis

Per 10 µL of RNA (1 µg total RNA) a total of 100 pmol oligo dT primers were added. The mix was heated to 70 ℃ for 5 min and cooled on ice afterwards. 8 µL of a mix consisting of 2 µL deoxyribonucleotide triphosphates (Promega, Madison, WI, USA), 4 µL 5× RT-buffer, 0.5 µL reverse transcriptase (Promega, Madison, WI, USA) and 1.5 µL RNase-free water were added. The reverse transcription was performed at 37 ℃ for 60 min and the reverse transcriptase was inactivated at 70 ℃ for 10 min. cDNA was stored at −20 ℃ until further analysis.

### 2.6. Quantitative Realtime PCR

To verify the relative gene expression in response to abiotic stress treatment a quantitative real time PCR (qRT-PCR) was performed on a LightCycler® 480 II operated with LightCycler® 480 SW 1.5.1.62 software (Roche Diagnostics Deutschland GmbH, Mannheim, Germany). GoTaq® qPCR Master Mix (Promega GmbH, Mannheim, Germany) was used in a total reaction volume of 10 µL according to manufacturers instruction using white 384-well plates (Sarstedt AG & Co. KG, Nümbrecht, Germany) and sealed with LightCycler® 480 Sealing Foil (Roche Diagnostics Deutschland GmbH, Mannheim, Germany). The qRT-PCR program was as follows: pre-denaturation: 180 s at 95 ℃, 45 cycles of: 15 s at 98 ℃, followed by 20 s at 58 ℃, and 20 s at 72 ℃, and final elongation: 180 s at 72 ℃. The melting curve was performed in the temperature range from 60 ℃ to 95 ℃ with an increase of 0.02 ℃/s.

Oligonucleotide primer pairs were designed using sequences with high read abundancies for stress treatments coming from the artificial transcriptome (reference genome: *C. annuum* cv. Zunla-1 [56]). Original sequences were first checked for similarities with the described known genes using BLAST. In case of full matches, the respective description is indicated in the supplementary Table S2. Otherwise, the artificial identifier starting with "MYSTRG" is used (see supplementary Table S2).

For analysis of the qRT-PCR data the ‘second derivative maximum method’ was performed with the LightCycler® 480 SW 1.5.1.62 software to calculate the crossing point (Cp) values that are related to the maximal acceleration of fluorescence. Additionally, cDNA of all samples of one species or ecotype were pooled to obtain a standard curve for determination of primer efficiency. The used dilutions were: 1:1, 1:10, 1:50, 1:100, 1:250 and 1:500.

Data obtained from qRT-PCR were analyzed with REST-384© developed by Pfaffl [67] relative to three reference genes: EIF5A2 (AY484392.1), Ubi3 (AY486137.1) and GAPDH (AJ246013.1) (see supplementary Table S2). Significance is evaluated by a Pair Wise Fixed Reallocation Test©, performing 10.000 reallocations, implemented in REST 384©.

### 2.7. Extraction of Metabolites

The same samples used for RNASeq analysis were also used to extract metabolites using 80% methanol (HPLC grade) in a final concentration of 125 mg/mL methanol. The samples were immediately mixed for 20 s, kept on ice and in the dark. Sonification for 20 min on ice was followed by 45 min centrifugation at 4 ℃ with full speed. The supernatants were evaporated in a SpeedVac at 60 ℃ for about 1 hour and stored at −80 ℃ until untargeted metabolite analysis was done.

### 2.8. Untargeted Metabolite Screening via QToF MS

For untargeted metabolomics, dried samples were solved using 1 µL 50% methanol per mg sample powder. A total of 10 µL of these extracts were diluted with 40 µL ultra-pure H_2_O and filtered through a PVDF membrane with 0.2 µm pores. 3 µL of each sample was injected onto a reverse-phase UPLC Cortecs C18 column (2.1 × 150 mm, 1.6 µm, Waters, Eschborn, Germany), using a binary Acquity UPLC I-class system running a gradient of mobile phases A (0.1% formic acid in water) and B (0.1% formic acid in acetonitrile) at a flow rate of 0.35 mL min^−1^ with the following program: 0 min 10% buffer B; 1 min 12% B, 10 min 32% B, 10.05 min 80% B, 11.95 min 80% B, 12 min 10% B, end at 16 min 10% buffer B. The column temperature was maintained at 45 ℃ throughout the run and sample temperature was constantly kept at 7 ℃. The column eluate was introduced directly into the mass spectrometer by electrospray ionization (ESI).

Mass spectrometric analysis was performed on a Vion IMS-Q-Tof (Waters, Eschborn, Germany) operating in both positive and negative mode. The capillary voltage was set to 1 kV (positive mode) or 0.8 kV (negative mode) and cone voltage of 20 V. The desolvation gas flow was set to 900 L h^−1^ and the desolvation temperature was set to 400 ℃. The cone gas flow was 50 L h^−1^, and the source temperature was set to 120 ℃. Accurate mass was maintained by introduction of Lock-Spray interface of Leucine-enkephalin (556.2771 [M + H]^+^ or 554.2615 [M − H]^−^) at a concentration of 50 pg µL^−1^ in 50% ACN and a rate of 10 µL min^−1^ (scan time 0.5 s, interval 20 s, scans to average 3). Mass signals were acquired in continuum mode from 50 to 900 mass-to-charge ratio (*m*/*z*) in High Definition MSe mode and 0.3 s scan time using the Unifi software package (Waters, Eschborn, Germany). Data analysis of raw mass spectrometric data was carried out using the software Progenesis QI (Waters, Eschborn, Germany). The full list of detected mass features in positive and negative mode can be found in supplementary Table S2.

### 2.9. Multi-Omics Analysis

PCA was carried out on metabolome profiles for all samples of each species to investigate the effect of different treatments and time points on each profile. Datasets were first transformed, using centered log ratio function “clr”, and scaled, using scale function, and then principle component analysis was performed using “prcomp” function.

**Figure 1:**
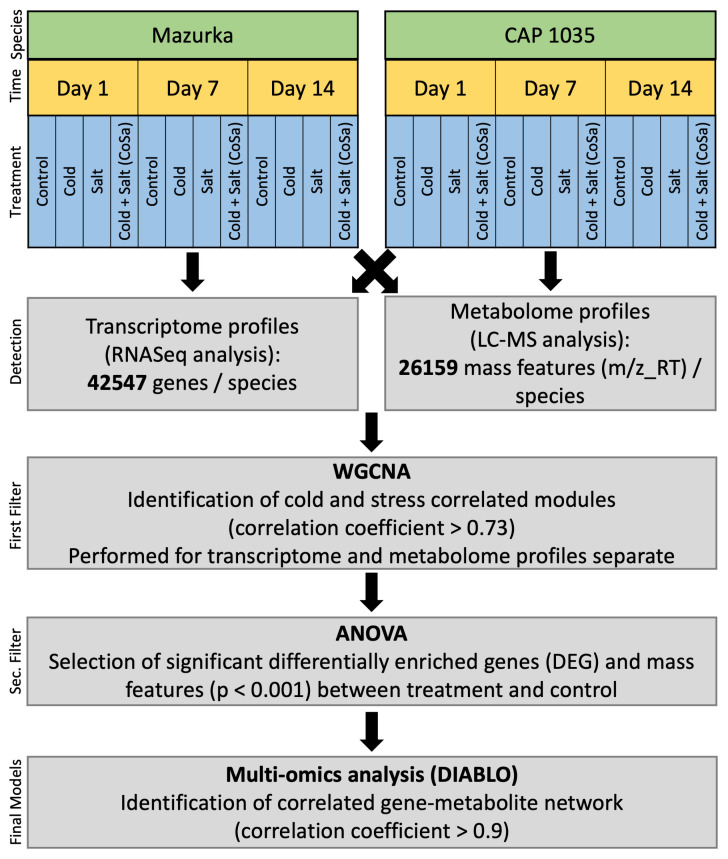
Workflow to explain the experimental design and pipeline that was used to detect key genes and metabolites related to abiotic stress.

In addition, transcriptome and metabolome profiles were analyzed together to identify correlated key omics variables for abiotic stress. Our dataset was divided into six different groups according to species and harvest time point. Each group contains the transcriptome and metabolome profiles of all treatments and the respective control at these harvest time points. Figure 1 and Table 1 explains the workflow and characteristics of our groups.

Further, for each group, weighted cluster analysis (WGCNA) was first performed separately on transcriptome and metabolome profiles to identify modules that are correlated with the abiotic stresses (minModuleSize = 30 and mergeCutHeight = 0.25, see also above) [66]. Only genes and mass features from highly correlated modules (correlation ≥ 0.73) were selected for the next step. An ANOVA analysis was performed to identify genes and mass features that differ significantly from the untreated samples (p≤0.001).

Finally, for each group, a separate DIABLO analysis (Data Integration Analysis for Biomarker discovery using a Latent component method for Omics studies) [51] was performed on the selected genes and mass features (see Table 1), using the “mixOmics”-package [68].

DIABLO is a multivariate dimension method that can correlate information between multiple datasets measured on different omics platforms. Each one of our DIABLO analyses contains 2 blocks: transcriptome profiles (Block 1) and metabolome profiles (Block 2), and the dummy matrix (Y) corresponding to the treatments (Cold, Salt, CoSa, and Control). The total number of components was set to 2 and a full design, with both blocks connected, was used for the comparison matrix. “Circosplot” function was performed and the correlation coefficient between all variables were calculated. Only variables that have at least a correlation value ≥0.9 were chosen for the following step. Genes that are annotated to secondary metabolites pathways by Mercator bins were chosen and their metabolites network were illustrated. All statistical analyses were performed using R [60].

### 2.10. Identification of Putatively Annotated Metabolites

Network analysis provided a list of compounds that are highly correlated with secondary metabolites genes in both species. To putatively annotate these unknown mass features, their mass to charge ratio (*m*/*z*) and the retention time were compared to these characteristics of known compounds that have been measured using the same LC-MS/MS approach. Only *m*/*z* matches with a deviation of up to 0.09, were chosen for a list of candidates of each mass feature. Then, candidates of this list were investigated for their presence and roles in the various secondary metabolite pathways of *Capsicum* sp. and the final list contains only matches that have been determined in any secondary metabolite pathway of *Capsicum* sp.

## 3. Results

### 3.1. Global Response of the Transcriptome Is Intensified over a Period of 14 Days

To investigate the abiotic stress response in the cultivated bell pepper line *Capsicum annuum* L. *var annuum* cv. Mazurka (Mazurka) and the chili species *Capsicum chinense* Jacq. cv. CAP 1035 (CAP 1035) an RNASeq analysis was performed using leaf samples harvested 1, 7 and 14 days after stress induction (for position of the harvested leaf, see Figure A1).

**Figure A2:**
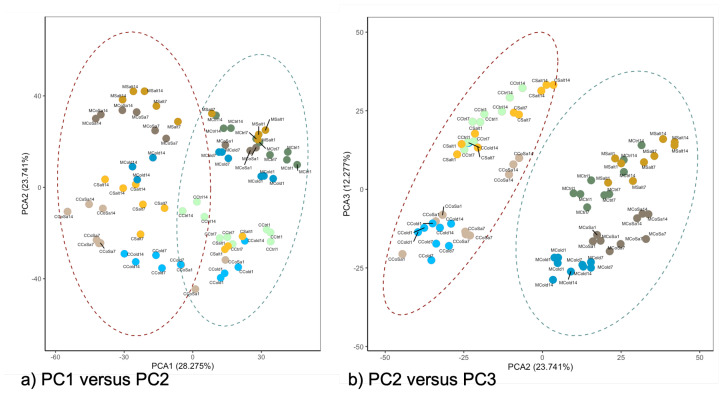
PCA from a combined analysis of Mazurka and CAP 1035. RNASeq reads were mapped to an artificial transcriptome comprising both transcriptomes coming from Mazurka (M) and CAP 1035 (C) (see also methods). Shown in (**a**) is the principal component 1 (PC1) versus principal component 2 (PC2), indicating that the highest variation in the combined analysis is coming from the harvest time point as indicated by the final number. In (**b**), the principal component 2 (PC2) is shown over principal component 3 (PC3), indicating that the next level of variation is influenced by the species (starting M versus starting C). Control samples (Ctrl) are indicated in green, cold samples (Cold) in blue, salt samples (Salt) in orange, and samples with a combination of salt and cold (CoSa) in brown.

In a first overall comparison, we mapped reads coming from both species to a combined artificial transcriptome (comprising both transcriptomes, see also methods) to get insights into whether the two species behave different or in principle similar. Our PCA showed a similar pattern (see also Figure A2) for both species. The highest variation in the combined analysis is coming from the harvest time point (see Figure A2a), while the next level of variation is influenced by the species (see Figure A2b).

**Figure 2:**
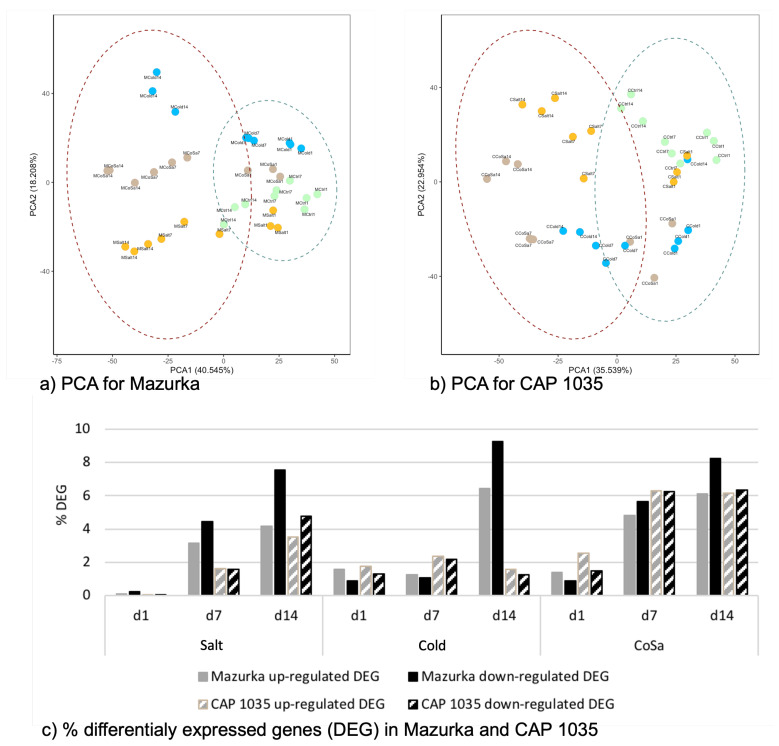
RNASeq analysis reveals overall similar behavior in the two pepper species. Principal component analysis for Mazurka (**a**) and CAP 1035 (**b**) indicating responsiveness to the harvest time points and stress treatments, shown by comparison of principal component 1 (PC1) versus principal component 2 (PC2). Control = green, Cold = blue, Salt = red, CoSa = light brown (**c**) Percentage of differentially expressed genes (DEG) in Mazurka (plain columns) and CAP 1035 (striped columns). Differentially expressed genes were obtained by global comparison of expression data for the respective stress treatment with the control sample. Percentage of down-regulated genes is shown in grey bars, and up-regulated genes in black bars, respectively. FDR < 0.01.

**Figure A3:**
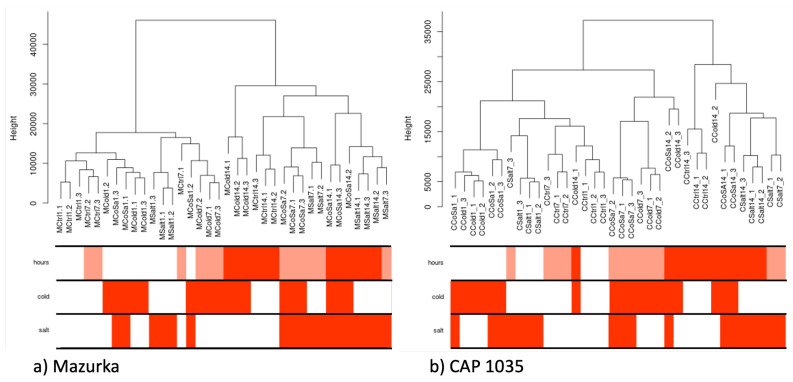
Dendrogram of trait cluster for Mazurka (**a**) and CAP 1035 (**b**) from single analysis approaches. RNASeq reads were used to perform a cluster network analysis (see also methods). The dendrogram shows what clusters will be formed according to the read abundance in combination with the single traits. Traits are indicated as harvest time point (hours), chilling temperature (Cold), or salt treatment (Salt). Combinatory stresses are indicated as CoSa. The number behind the stress indication reflects first the harvest time point (1, 7 or 14) followed by a number for the single RNASeq sample (1, 2 or 3).

Afterwards, we performed the analysis for each pepper cultivar separately. In this context, the proportion of variance for both species was found to be similar, so that the main difference between samples can be explained by PC1 (40.55% for Mazurka and 35.54% for CAP 1035) and PC2 (18.21% for Mazurka and 22.95% for CAP 1035). In addition, the PCA revealed that for both species samples harvested at the early time point (d1, in any combination) were grouped together with the control samples from all three harvest time points (see Figure 2a,b). Samples from later harvest timepoints form more distinct separate groups, with the exception of samples under chilling temperature at d7 in Mazurka, which stay closer to the control group, and samples from a combined stress at d7 in CAP 1035, which form a group with samples under chilling temperature alone. An additional hierarchical clustering revealed a similar pattern (see Figure A3). For Mazurka, one cluster comprises control samples from d1 and d7 together with samples from stress treatments at d1 and samples treated with chilling temperature at d7. A second cluster for Mazurka is formed by samples treated with salt or a combination of chilling temperature and salt at d7 and d14 together with control samples and samples grown under chilling temperature at d14 (see Figure A3a). For CAP 1035, three clusters can be observed with one cluster harboring all samples harvested at d1, control samples at d7, a single sample treated with salt at d7 and a single sample grown under chilling temperature at d14 (see Figure A3b). A second cluster is formed by all samples grown under chilling temperature or a combinatorial stress treatment at d7, in addition to a single sample grown under chilling temperature or a combinatorial stress treatment at d14. The third cluster is formed by all samples harvested at d14, besides the formerly indicated samples, in addition to two salt-treated samples at d7.

**Figure A4:**
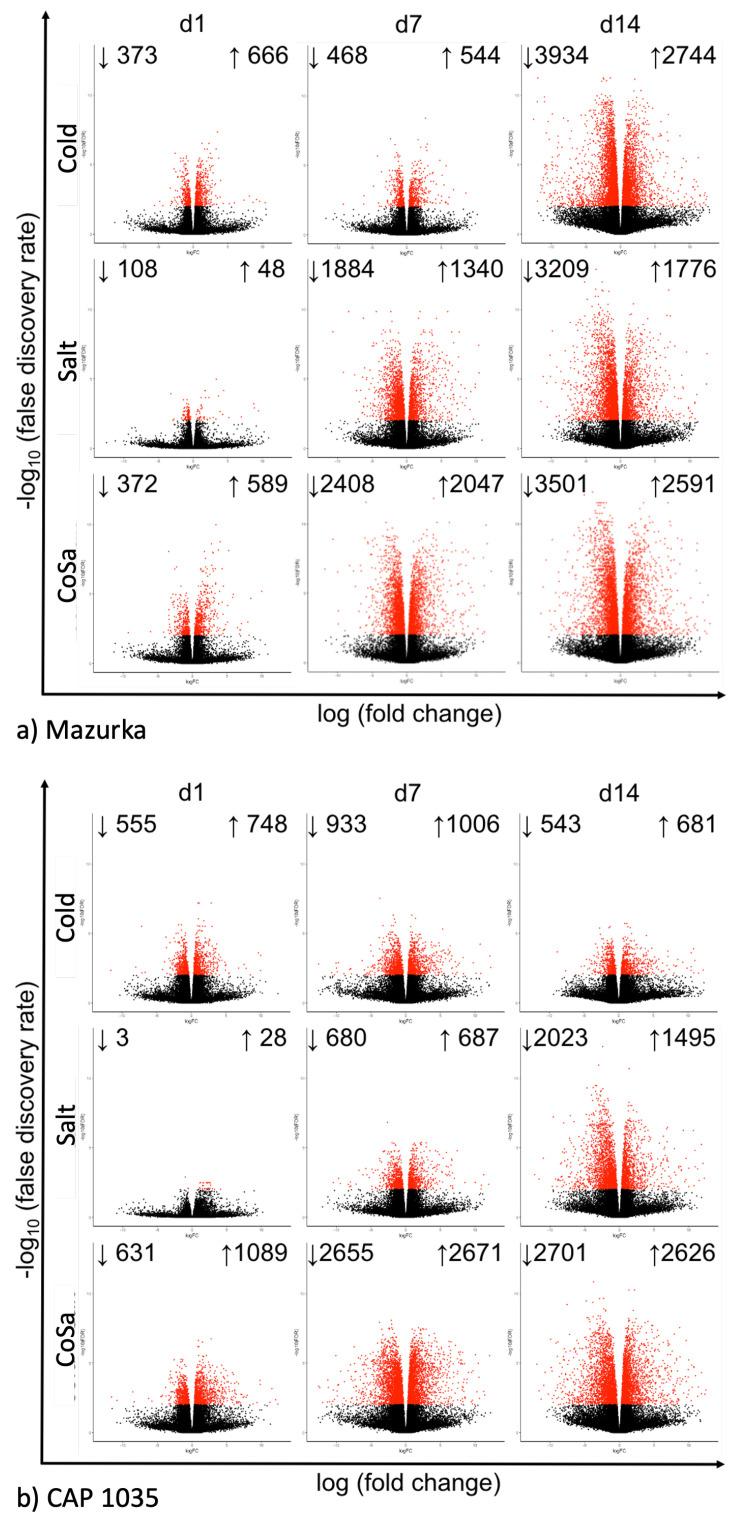
Volcano plots indicating the differentially expressed genes in Mazurka (**a**) and CAP 1035 (**b**). Plants were grown under different abiotic stress conditions (chilling temperatures: cold, watering with additional 200 mM NaCl (salt), and a combination thereof (CoSa) as indicated) on rockwool. The leaf after first branching was harvested at day 1 (d1), day 7 (d7) and day 14 (d14) (as indicated) and a transcriptome analysis was performed comparing the different stress conditions with the control sample from the respective harvest time point. The negative decadic logarithmic values (−log_10_) of the false discovery rate are plotted against the logarithmic values of the fold change for each condition in both species. Significantly different expressed genes are highlighted in red, and total numbers are indicated. FDR < 0.01.

**Figure A5:**
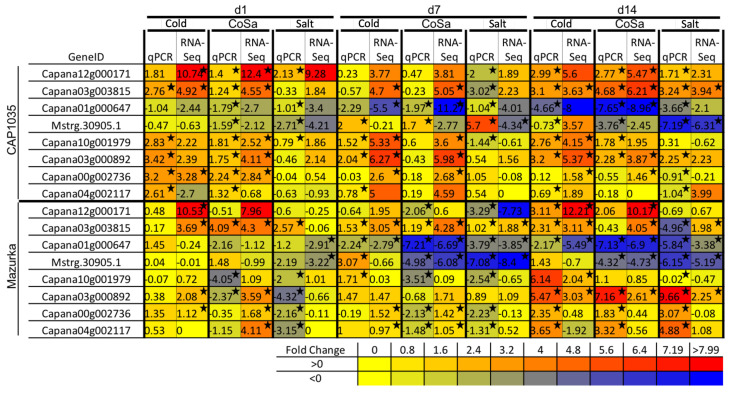
Verification of RNASeq data by quantitative realtime PCR. RNASeq data were verified using samples from an independent biological experiment performed within the same plant chambers under the same stress conditions as before. Mazurka and CAP 1035 were grown on rockwool for six weeks before applying chilling temperatures (Cold), 200 mM salt (Salt) or a combination thereof (CoSa). A total of 1, 7 and 14 days after treatment induction the expression of indicated genes was determined in 4 biological replicates per treatment. The results are expressed as log_2_(fold change compared to the control sample). Significance is indicated for *p* < 0.001 as asteriks, or an FDR < 0.01 for RNASeq data.

When checking the percentage of differentially expressed genes (DEG) by comparison with the control samples, it becomes obvious that the number of DEG increases from d1 to d14 when salt was applied (see Figure 2c and Figure A4). Watering with 200 mM NaCl (Salt) gave only little DEG after one day, while it had a strong effect after 14 days. Chilling temperature conditions alone (Cold) had a mild but early effect in both species with only a slight increase of DEG between d1 and d7 in both species. At d14 we found a significant enrichment of DEG for Mazurka but not CAP 1035. The combination of chilling temperatures and watering with salt (CoSa) seemed to have an additive effect for both species at d7. For the pungent species CAP 1035, we do observe the additive effect also at d14, but not for the sweet pepper Mazurka. RNASeq data was confirmed by a quantitative real time PCR with samples from an independent biological experiment. Gene expression was determined for a random subset of eight genes with significant enrichment or suppression in accordance with the RNASeq data (see Figure A5).

We conclude from our observation, that pepper plants respond to the treatment of chilling temperature, salt or a combination thereof, with the strongest transcriptomic response at d7 and d14 under a combinatorial stress treatment in both species.

### 3.2. Differences in the Transcriptional Stress Response of the Two Species

We performed a detailed RNASeq analysis to get insights into what metabolic pathways were induced or repressed by the abiotic stress treatment. First, we performed an overrepresentation analysis (ORA) to find significantly enriched categories using the MapMan ontology [69,70,71] in the subset of up- or down-regulated genes, respectively.

With the aim to link observed expression changes to potential biological responses, transcripts will be grouped in classes (or bins in case of PageMan), followed by an analysis if transcripts (within the bin) respond in a concerted way. In general, the overrepresentation of a category indicates a direct effect of the applied abiotic conditions (either induction for up-regulated genes or repression for down-regulated genes). In contrast, the ORA will result in an underrepresentation, if the expression of the transcripts within a given bin is not affected by the applied conditions.

**Figure 3:**
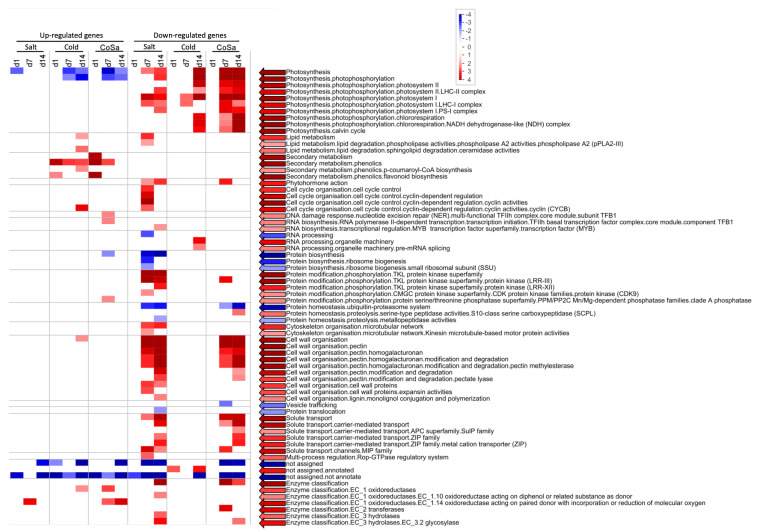
Overrepresentation analysis (ORA) performed for up- or down-regulated DEG of Mazurka. Plants were grown under different abiotic stress conditions (Salt, chilling temperatures: Cold, and combinations thereof: CoSa) on rockwool. The leaf after first branching was harvested and a transcriptome analysis was performed comparing the different stress conditions with the control sample. The over- or underrepresentation of indicated Mercator [71] bins were calculated separately for up- (left column) and down-regulated genes (right column) using a Fisher test [65] in combination with a multiple testing correction as performed by Benjamini and Hochberg [72]. Overrepresented bins are shown in red and underrepresented bins in blue, respectively.

**Figure 4:**
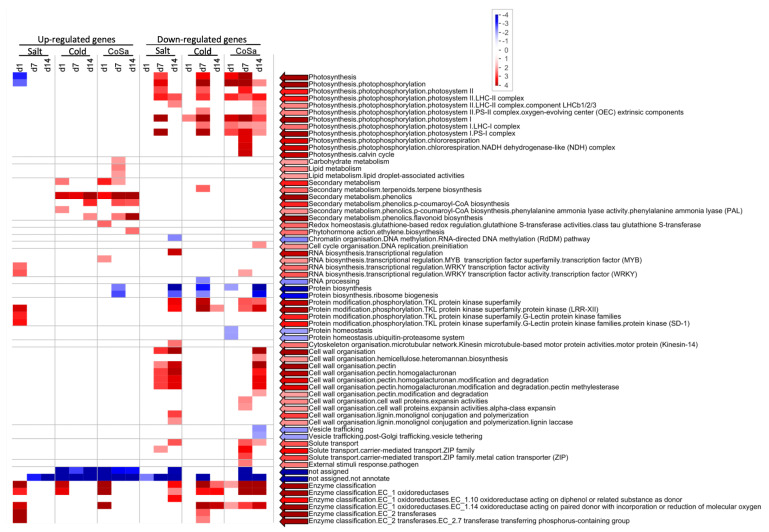
Overrepresentation analysis (ORA) performed for up- or down-regulated DEG of CAP 1035. Plants were grown under different abiotic stress conditions (Salt, chilling temperatures: Cold, and combinations thereof: CoSa) on rockwool. The leaf after first branching was harvested and a transcriptome analysis was performed comparing the different stress conditions with the control sample. The over- or underrepresentation of indicated Mercator [71] bins were calculated separately for up- (left column) and down-regulated genes (right column) using a Fisher test [65] in combination with a multiple testing correction as performed by Benjamini and Hochberg [72]. Overrepresented bins are shown in red and underrepresented bins in blue, respectively.

Overall, more overrepresented categories were revealed for down-regulated genes, whereas up-regulated genes were strongly overrepresented in the main category of secondary metabolism for both species (see Figure 3 and Figure 4).

For Mazurka and CAP 1035, **up-regulated genes** were overrepresented in **secondary metabolism** pathways especially from the early time point (d1) on for treatments involving chilling treatment. Salt treatment alone did not induce an overrepresentation of up-regulated genes in the secondary metabolism (see Figure 3 and Figure 4).

Phenolics biosynthesis was enriched by chilling temperatures (Cold)and the combination treatment (CoSa) at all harvest time points, with the exception of d14 in the combined stress in Mazurka (see Figure 3 and Figure 4). In Mazurka, only at d14 under chilling temperatures, the p-coumaroyl-CoA biosynthesis was also shown to be enriched for up-regulated genes, whereas in CAP 1035 phenylalanine ammonia lyase (PAL) is enriched at d1 by chilling and p-coumaroyl-CoA biosynthesis in both treatments involving chilling at d7 (Cold)and d14 (both). The specific flavonoid synthesis was enriched early at d1 for cold and CoSa in Mazurka and later in CAP 1035 (Cold: d14, CoSa: d7 and d14) in both treatments involving chilling, which coincides with the p-coumaroyl-CoA biosynthesis.

Besides, pathways belonging to lipid degradation, as well as cyclin-dependent regulating pathways and cell wall organisation in general were overrepresented in up-regulated genes at d14 under chilling temperatures in **Mazurka**. Also, pathways of the nucleotide exchange repair (NER), initiation of RNA-polymerase II transcription and protein phosphorylation through the serine/threonine phosphatase superfamily were overrepresented in up-regulated genes at d7 under combinatorial stress (CoSa) in Mazurka (see Figure 3).

In **CAP 1035**, pathways belonging to carbohydrate metabolism and lipid droplet-associated activities were enriched in up-regulated genes at d7 under chilling temperatures. Furthermore, regulating pathways involving WRKY transcription factor and protein phosphorylation by tyrosine kinase-like (TKL) protein kinase superfamily were overrepresented at d1 under salt treatment (see Figure 4). MYB transcription factor regulated RNA biosynthesis was specifically enriched at d1 under a combinatorial stress. Glutathione S-transferase activities were overrepresented in up-regulated genes at d1 and ethylene biosynthesis at d14 under combinatorial stress treatment.

With regard to **down-regulated genes**, pathways belonging to **photosynthesis** were depleted by any stress treatment at d7 and d14 for both Mazurka and CAP 1035 (see Figure 3 and Figure 4). However, only CAP 1035 showed an early response for some of the photosynthesis pathways when the combinatorial stress was applied (at d1 CoSa). Especially phosphorylation of photosystem I and II were affected as well as chlororespiration and the calvin cycle. In general, Mazurka had more photosynthesis pathways repressed at d14 than CAP 1035. Both species showed an expanded response of genes in photosynthesis pathways at every harvest point under combined stresses.

In addition under **chilling temperature**, pathways of pre-mRNA splicing were down regulated at d14 in Mazurka, and protein phosphorylation by the TKL protein kinase superfamily were depleted at d7 and d14 in CAP 1035.

Often we observed a similar pattern of overrepresentation for plants under **salt stress or a combination of salt stress and chilling temperatures** in both species (see Figure 3 and Figure 4). In the following, only the differences will be highlighted. In Mazurka, **lipid degradation** by phospholipase A2 was overrepresented in down-regulated genes at d7 under salt stress, while none of these pathways seemed to be depleted in CAP 1035. Also pathways belonging to **phytohormone action** were overrepresented in down-regulated genes at d7 and d14 under salt treatment and at d7 under the combinatorial stress in Mazurka, which was not the case for CAP 1035. Salt stress also affected the cyclin dependent **cell cycle** regulation at d7 in Mazurka, while pathways involved in preinitiation of DNA replication were depleted at d14 in CAP 1035 under the combination of salt and chilling temperatures. **RNA biosynthesis** in general was down-regulated at d14 under salt treatment in CAP 1035, and transcriptional regulation via WRKY transcription factors were depleted at d7 under a combination of salt and chilling temperatures. In Mazurka, transcriptional regulation via MYB transcription factors was depleted at d14 under salt treatment. **Protein phosphorylation** by TKL protein kinase superfamily was down-regulated at d7 and d14 under salt stress and at d7 under the combinatorial stress in Mazurka, while in CAP 1035 those pathways were depleted at d14 under salt stress and at d7 and d14 under the combination of salt and chilling temperatures. The microtubular network of the **cytoskeleton** was down-regulated under salt stress at d7 and d14 in Mazurka and at d14 in CAP 1035. Pathways of the **cell wall** regulation belonging to pectin modification and degradation were highly affected in the down-regulated genes at d7 and d14 in Mazurka after both salt treatment or a combination, and in CAP 1035 at d7 and d14 after salt treatment and at d14 after a combination. Expansin activities were depleted at d7 under salt stress in Mazurka and at d7 under a combinatorial stress in CAP 1035, while monolignol conjugation and polymerisation via lignin laccase were depleted at d14 in both species after salt treatment. Carrier-mediated **solute transport** was down-regulated at d14 under both salt stress and a combinatorial stress in Mazurka, while transport via major intrinsic protein (MIP) channels were depleted at d7 and d14 under salt stress and at d7 under the combination. In CAP 1035, only carrier-mediated solute transport was overrepresented in down-regulated genes at d7 and d14 under both salt stress and a combinatorial stress. Pathways belonging to the Rho of plants (Rop) GTPase regulatory system, which controls a multitude of cellular mechanisms [73] (part of a **multi-process regulation**), were depleted at d7 under salt stress in Mazurka, but not affected in CAP 1035. Pathways of an **external stimuli response** to pathogens were down-regulated at d7 under a combination of salt stress and chilling temperatures in CAP 1035.

In total, we observed a repression of photosynthesis in both species under any condition. Further cell wall organization and solute transport are as well repressed in both species under salinity and a combination of chilling temperature and salt. For secondary metabolism we noticed an induction under chilling temperature and the combinatorial stress in both species. For all other annotated biological functions, the pattern of induction or repression was less obvious and/or not correlated between the species. To get further insights we performed a WGCNA with the transcriptomic data.

### 3.3. Differences between Early and Late Response Detected by Co-Expression Networks of the Transcriptome

With the help of a WGCNA [66] (performed for each species and each harvest time point alone), we identified co-expression networks (modules) related to a specific stress (binary trait). The network matrix was built by checking for differences between the control group and the stress treatments. In addition, single stresses were set as binary trait.

**Figure A6:**
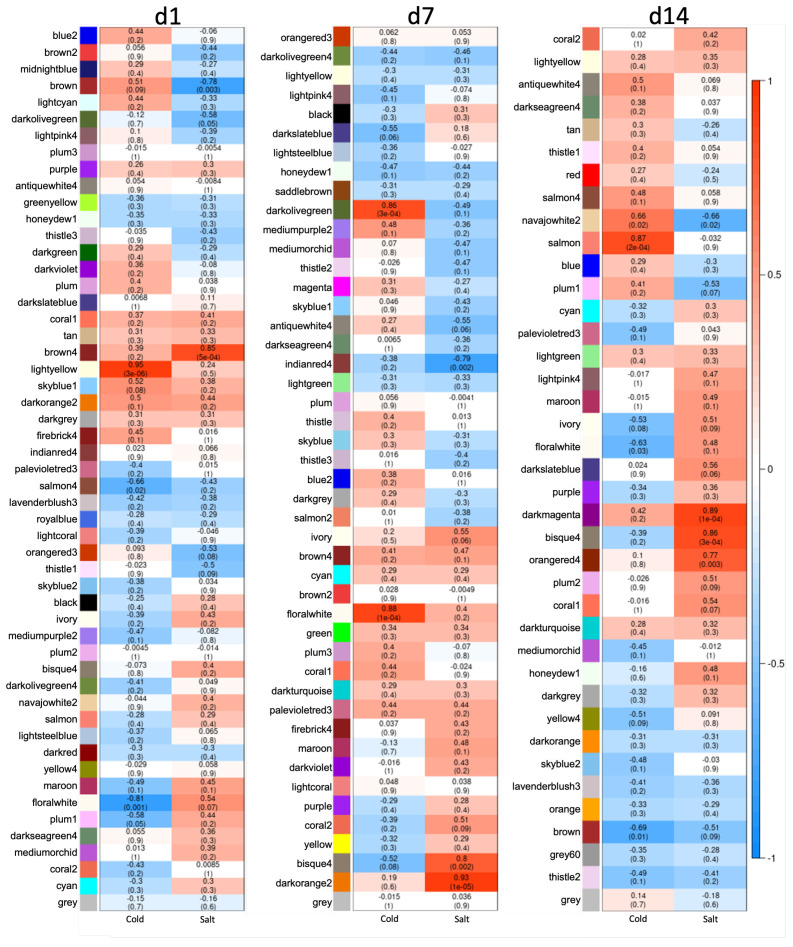
Module-trait correlation for the transcriptome of Mazurka. RNASeq data was used to perform a WGCNA. Shown here is the relationship for each identified cluster with the single stress chilling temperature (Cold), or salt (Salt) as indicated at d1, d7 or d14. On the y-axis, all identified modules are listed with their respective names. Color and number indicate the correlation between module and binary trait for eigengenes and numbers in brackets the *p*-Value for significance.

**Figure A7:**
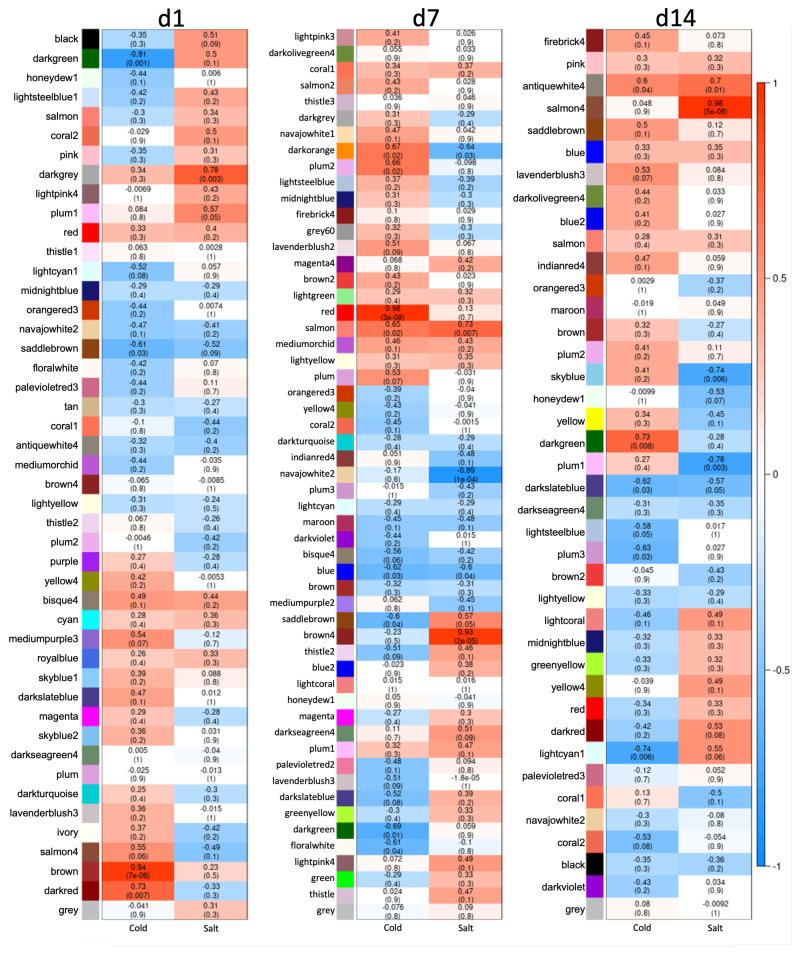
Module-trait correlation for the transcriptome of CAP 1035. RNASeq data was used to perform a WGCNA. Shown here is the relationship for each identified cluster with the single stress chilling temperature (Cold), or salt (Salt)as indicated at d1, d7 or d14. On the y-axis, all identified modules are listed with their respective names. Color and number indicate the correlation between module and binary trait for eigengenes and numbers in brackets the *p*-Value for significance.

For each module the correlation for all harboring genes with the binary trait (chilling temperatures or salt treatment) was calculated. In total, for the cultivated pepper line Mazurka we identified 53 modules at d1, 46 modules at d7 and 39 module at d14 (see Figure A6). For the wild-relative CAP 1035 we identified 46 modules at d1, 55 modules at d7 and 40 modules at d14 (see Figure A7). Modules with a correlation of 0.73 and higher (later called significant modules) for a specific binary trait were investigated further to identify characteristics among those genes.

**Figure A8:**
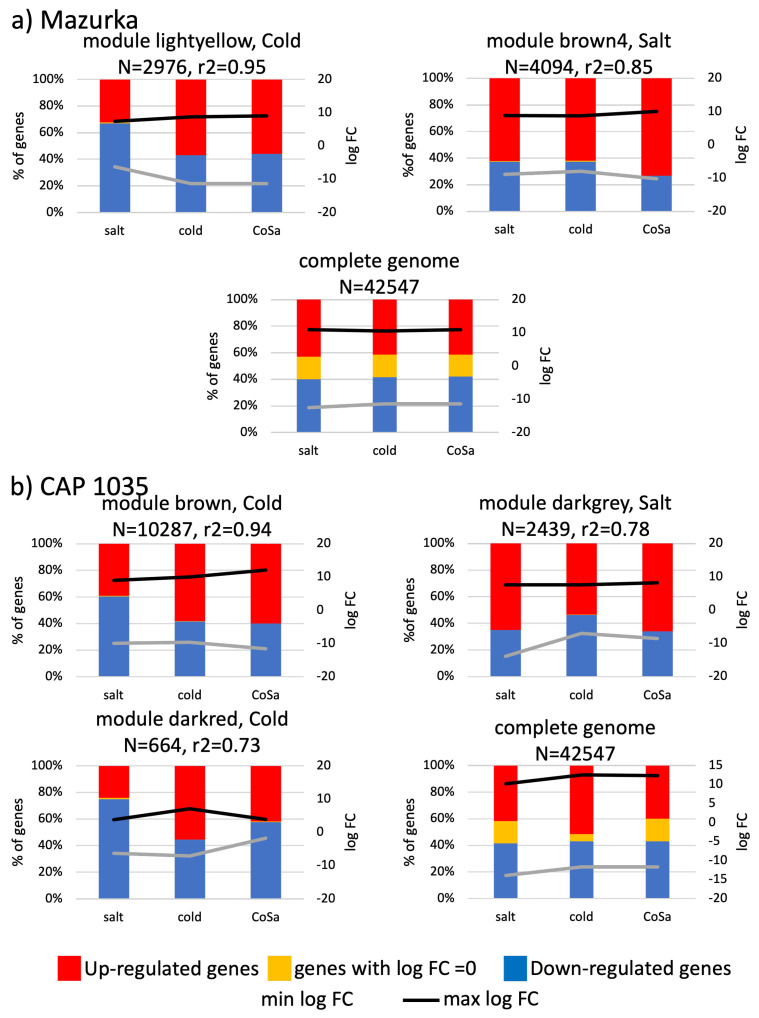
Characteristics of modulesfrom the transcriptome with a high correlation for one binary trait shown for Mazurka (**a**) and CAP 1035 (**b**) at d1. RNASeq data was used to perform a WGCNA. Modules with a correlation of 0.73 and higher were investigated for the presence of up- (red) or down-regulated (blue) genes. Genes having a log_2_ FC = 0 are indicated in yellow. The absolute maximal log_2_ FC (black line) and minimal log_2_ FC (grey line) are indicated. Beneath the module name the number of genes within the module is shown. The graph on the bottom shows data for the whole genome.

**Figure A9:**
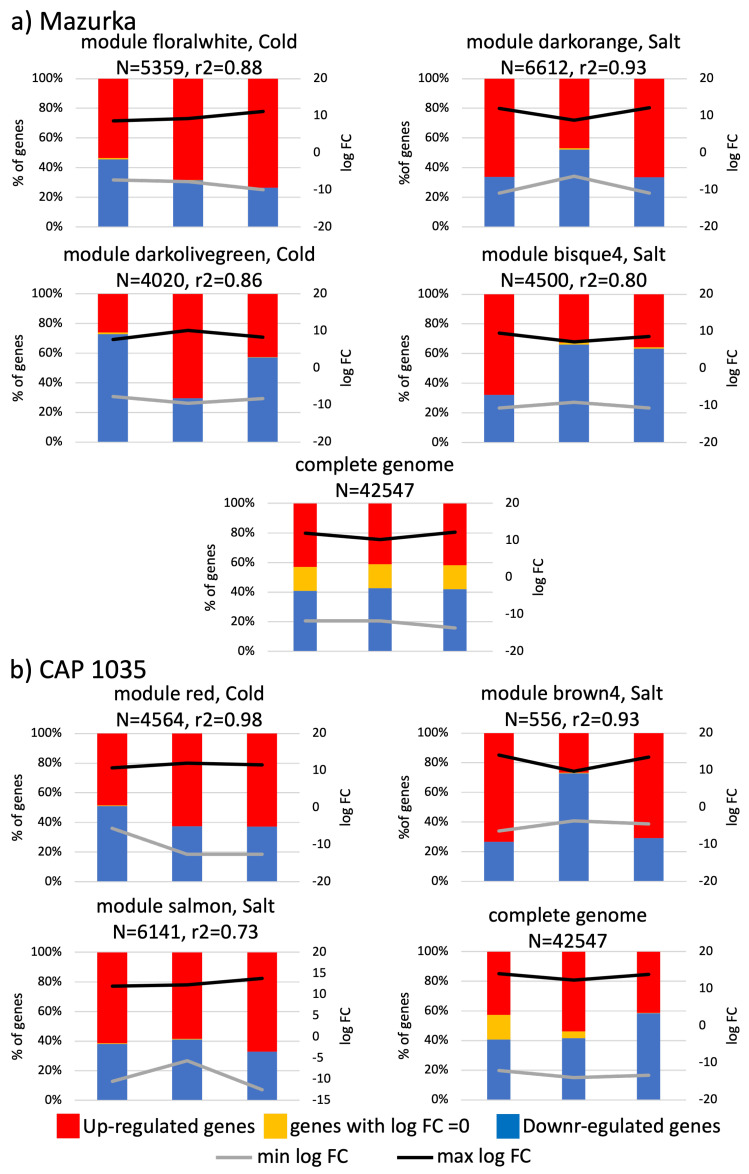
Characteristics of modules from the transcriptome with a high correlation for one binary trait shown for Mazurka (**a**) and CAP 1035 (**b**) at d7. RNASeq data was used to perform a WGCNA. Modules with a correlation of 0.73 and higher were investigated for the presence of up- (red) or down-regulated (blue) genes. Genes having a log_2_ FC = 0 are indicated in yellow. The absolute maximal log_2_ FC (black line) and minimal log_2_ FC (grey line) are indicated. Beneath the module name the number of genes within the module is shown. The graph on the bottom shows data for the whole genome.

**Figure A10:**
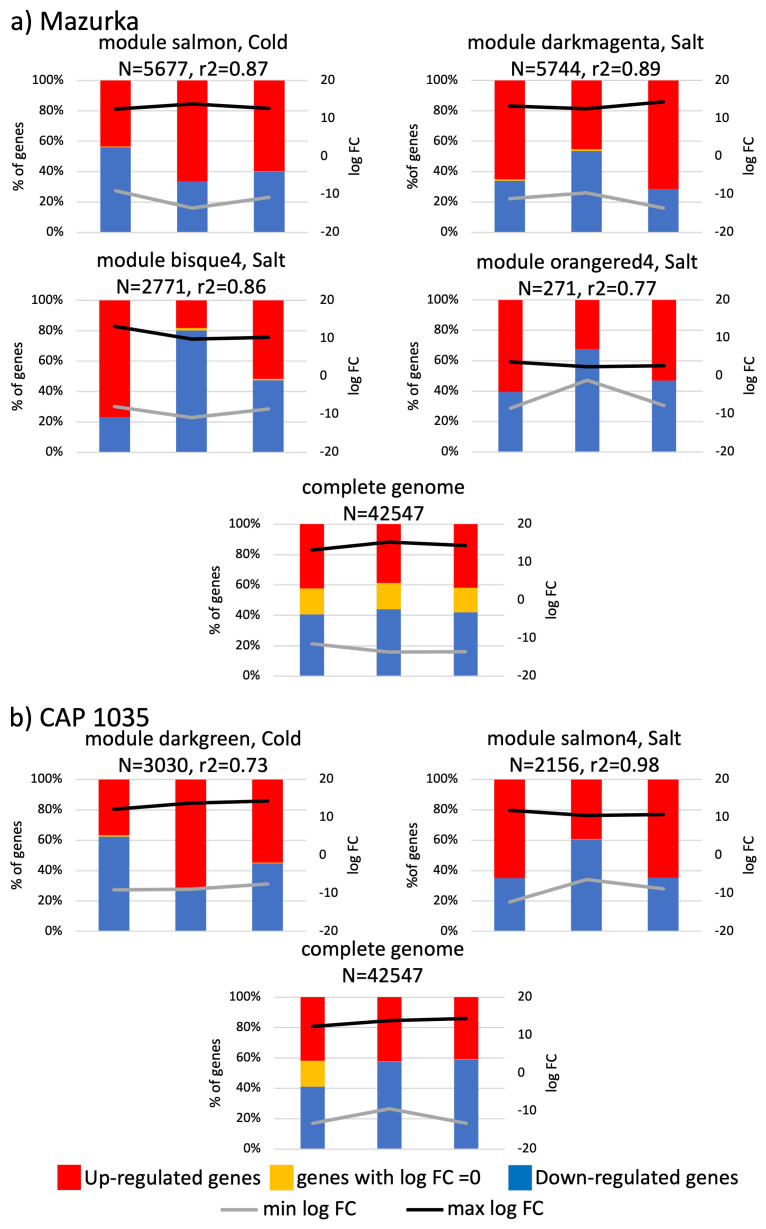
Characteristics of modules from the transcriptome with a high correlation for one binary trait shown for Mazurka (**a**) and CAP 1035 (**b**) at d14. RNASeq data was used to perform a WGCNA. Modules with a correlation of 0.73 and higher were investigated for the presence of up- (red) or down-regulated (blue) genes. Genes having a log_2_ FC = 0 are indicated in yellow. The absolute maximal log_2_ FC (black line) and minimal log_2_ FC (grey line) are indicated. Beneath the module name, the number of genes within the module is shown. The graph on the bottom shows data for the whole genome.

First, for all genes within a significant module we checked the presence of up- and down-regulated genes for each applied stress condition (see Figure A8, Figure A9 and Figure A10). Data from the whole genome was used as a control group for comparison.

In **Mazurka** we investigated for d1 the significant modules lightyellow (related with chilling temperatures) and brown4 (related with salt) (see Figure A8). They included 2976 and 4094 genes, respectively. For d7 we examined the modules floralwhite and darkolivegreen (both related with chilling temperature) as well as darkorange and bisque4 (both related with salt), harboring between 4020 and 6612 genes per each module (see Figure A9). For d14 we reviewed module salmon (5677 genes, related with chilling temperatures) and the modules darkmagenta (5744 genes), bisque4 (2771 genes) and orangered4 (271 genes) (all related with salt) (see Figure A10).

For all significant modules, we found an enrichment for up-regulated genes under the respective trait as single stress treatment or in a combination. In addition, the absolute value of maximal and minimal log FC increased, respectively, for the correlating traits when compared with other conditions, indicating that the altered expression responses will have a higher biological relevance and impact on the stress response.

For **CAP 1035**, we observed a similar pattern for the significant modules brown (10,287 genes) and darkred (664 genes) (both related with chilling temperature), as well as darkgrey (2439 genes, related with salt) (see Figure A8) at d1. For d7 we considered the modules red (4564 genes, related with chilling temperature), brown4 (556 genes) and salmon (6141 genes) (both related with salt) for further analysis (see Figure A9). In addition, for d14 we analyzed modules darkgreen (3030 genes, related with chilling temperature) and salmon4 (2156 genes, related with salt) (see Figure A10). Still, we conclude, that the accumulated genes within the significant modules respond mainly to the respective trait.

To investigate what kind of **functional categories** will be represented by genes within the significant modules, we checked the respective Mercator bins. For this purpose, we collapsed the first 8 bins (namely Photosynthesis, Cellular respiration, Carbohydrate metabolism, Amino acid metabolism, Lipid metabolism, Nucleotide metabolism, Coenzyme metabolism and Polyamine metabolism) to a combined category of “Primary metabolism”. In addition, bins of Chromatin organization, Cell cycle organization, and DNA damage response are summarized in “Cell cycle and DNA”, RNA biosynthesis and RNA processing in “RNA”, Protein biosynthesis, Protein modification, and Protein homeostasis in “Protein”, Cytoskeleton organization and Cell wall organization in “Cytoskeleton and Cell wall”, Vesicle trafficking, Protein translocation, Solute transport and Nutrient uptake in “Transport” and all other bins with specific functions (besides “Secondary metabolism”) are summed in “External stimuli and divers processes”. “General annotation” reflects the categories Enzyme classification and not assigned/annotated, and “Unknown” describes the bin not assigned/not annotated.

We then checked in all significant modules the presences of aforementioned categories for all genes. We combined multiple modules, in case of two or three significant modules for one binary trait at a given harvest time point. As a control we checked for the present proportion of those bins in the whole genome (without performing a network cluster analysis).

**Figure 5:**
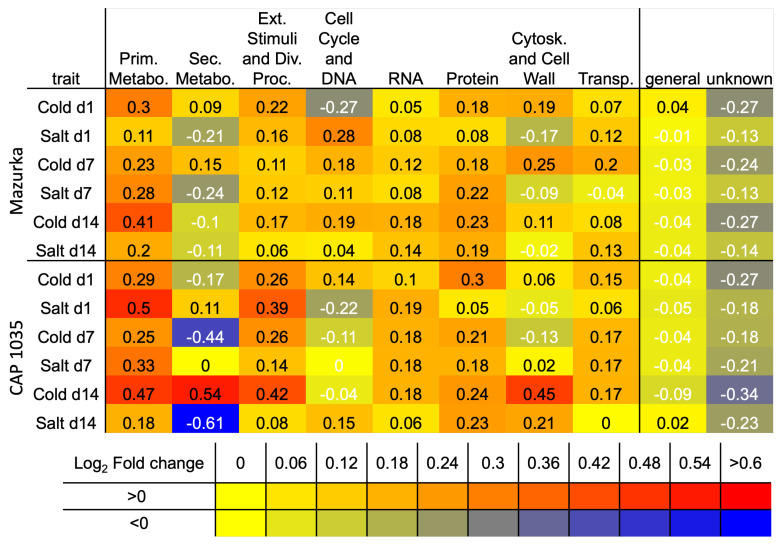
HeatMap of functional characteristics in significant modules of WGCNA for both species. Co-expression analysis (WGCNA, Ref. [66]) was performed for both transcriptomes individually with all samples from harvest time point as indicated. Within the significant modules, all genes were characterized and summarized according to their biological function of indicated Mercator bins [71]. Afterwards, the log2 (fold change) of significant genes between sample and control were calculated. Values >|0.25| were considered to be relevant.

**Figure A11:**
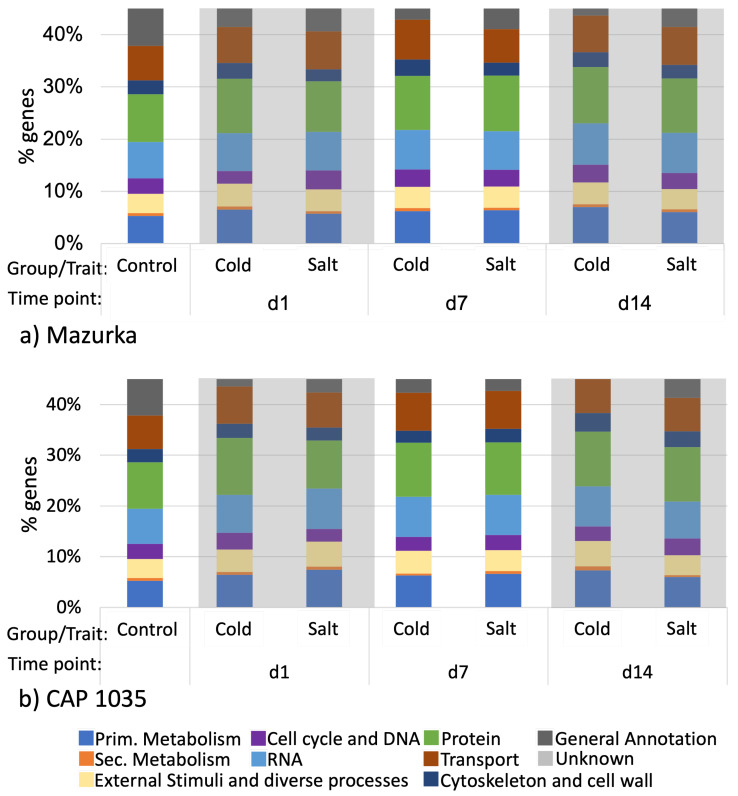
Functional characteristics of genes in significant modules of WGCNA for both species. Co-Expression analysis (WGCNA, Ref. [66]) was performed for both transcriptomes individually with all samples from the harvest time point as indicated (data for Mazurka is shown in (**a**), data for CAP 1035 in (**b**)). Within the significant modules, all genes were characterized and summarized according to their biological function of indicated Mercator bins [71]. Not shown are 55% of the genes. Those belong to the categories “General Annotation” and “Unknown”.

In both species, about 55% of all genes (in the whole genome, but also within the significant modules) were annotated to the general categories Enzyme classification and not assigned/annotated (summarized in “General annotation”, dark grey colored in Figure A11), or of unknown function (depicted as “Unknown”). For **Mazurka**, we found a slight enrichment of genes belonging to the primary metabolism at d1 and d14 in the significant modules related with chilling temperature (see Figure 5 and Figure A11), and the same was true for the significant modules related to salt at d7. At d1, we found an enrichment of genes belonging to the cell cycle and DNA damage response in the significant modules for salt stress (see Figure 5 and Figure A11a).

In **CAP 1035**, we examined a higher proportion of genes for the primary metabolism and responding to external stimuli in any significant module (see Figure 5 and Figure A11b) compared to the whole genome. Additionally, for significant modules related with chilling temperatures, we found a higher proportion of genes related with protein biosynthesis at d1 and cytoskeleton and cell wall function at d14. The proportion of genes related with secondary metabolism were enriched only at d14 in the significant module related with chilling temperature, while the proportion was reduced at d7 under chilling temperature and d14 under salt stress.

In any investigated significant module, the proportion of genes with unknown function or belonging to the general annotation were reduced, accompanying the slight enrichment of most other categories in both species.

We conclude from this analysis, that significant modules harbor a higher percentage of genes correlating with the indicated trait. Following, we will use this set of genes (coming from the transcriptome analysis) and correlate them with metabolomic data to decipher the underlying network.

### 3.4. Metabolome Analysis

**Figure 6:**
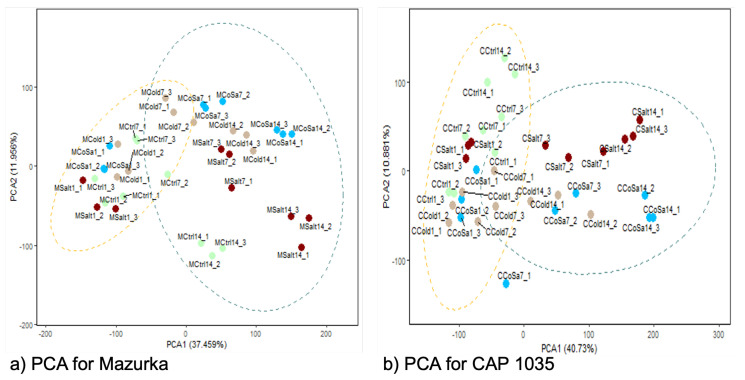
Metabolome analysis reveals overall similar behavior in the two pepper species. Metabolites were extracted from harvested material of day 1 (d1), day 7 (d7) and day 14 (d14) as described in the methods. (**a**,**b**) Principal component analysis of detected mass features for Mazurka (**a**) and CAP 1035 (**b**) indicating responsiveness to the harvest time points and stress treatments, shown by comparison of principal component 1 (PC1) versus principal component 2 (PC2). Control = green, cold = light brown, salt = red, CoSa = blue.

To investigate the effect of abiotic stress on the metabolome profiles in both species, PCA analysis of the detected mass features was carried out. The first two principal components (PCs) can explain approximately 50% of the variation in the metabolome profiles of both species (PC1: 37.46% for Mazurka and 40.73% for CAP 1035; and PC2: 11.96% for Mazurka and 10.88% for CAP 1035), with principal component 1 standing for the harvest time point and principal component 2 summarizing different stress treatments. In addition, PCA in both species showed the effect of abiotic stresses after 14 days on the metabolome profiles, e.g., samples of stressed leaves were clearly separated from untreated samples (see Figure 6a,b). Further, samples of each treatment were also separated from each other. In Mazurka, the effect of different abiotic stresses was noticed after only 7 days of treatment, where stressed samples were separated from the untreated samples (see Figure 6a). However, in CAP 1035 the effect of salt and the combination treatment were detected after 7 days while cold stress affected the metabolome profile only after 14 days (see Figure 6b).

We observed an increasing number of mass features affected by any of the stress treatments over the harvest period while analyzing the mass to charge ratio (*m*/*z*), retention time and peak area of all detected mass features and comparing them with the control (see Table 2). In general, we detected more accumulated mass features than compounds with reduced abundance. For Mazurka, a considerable increase of affected mass features was detected between d1 and d7, while for CAP 1035 this behavior is visible between d7 and d14. CAP 1035 had less affected mass features in total when compared with Mazurka at d1 and d7 (Mazurka: d1—352, d7—2040; CAP 1035: d1—70, d7—956), but the final number of total affected mass features at d14 was higher in CAP 1035 (4691) than in Mazurka (3385).

Our results suggest that both species accumulate metabolites due to our stress treatments, with CAP 1035 showing slightly more induced metabolites than the cultivated Mazurka. However, the absolute number of induced metabolites is increasing for both over time.

### 3.5. DIABLO Analysis

To identify a multi-omics signature that discriminates treated and untreated samples, six different groups, three for Mazurka (d1, d7 and d14) and three for CAP 1035 (d1, d7 and d14), were analyzed using multi-omics analysis (DIABLO). Only genes and mass features that are correlated to a stress trait (identified by WGCNA, correlation > 0.73), and that differed significantly between treatment and control (identified by ANOVA, *p* < 0.001) were used for the DIABLO analysis (see Figure 1). In total, for the cultivated pepper line, Mazurka 437 genes and 352 mass features were identified at d1, 2320 genes and 2040 mass features at d7 and 5623 genes and 3385 mass features at d14. For the wild-relative CAP 1035, 1489 genes and 70 mass features were identified at d1, 3413 genes and 956 mass features at d7 and 1818 genes and 4691 mass features at d14 (see Table 1).

**Figure 7:**
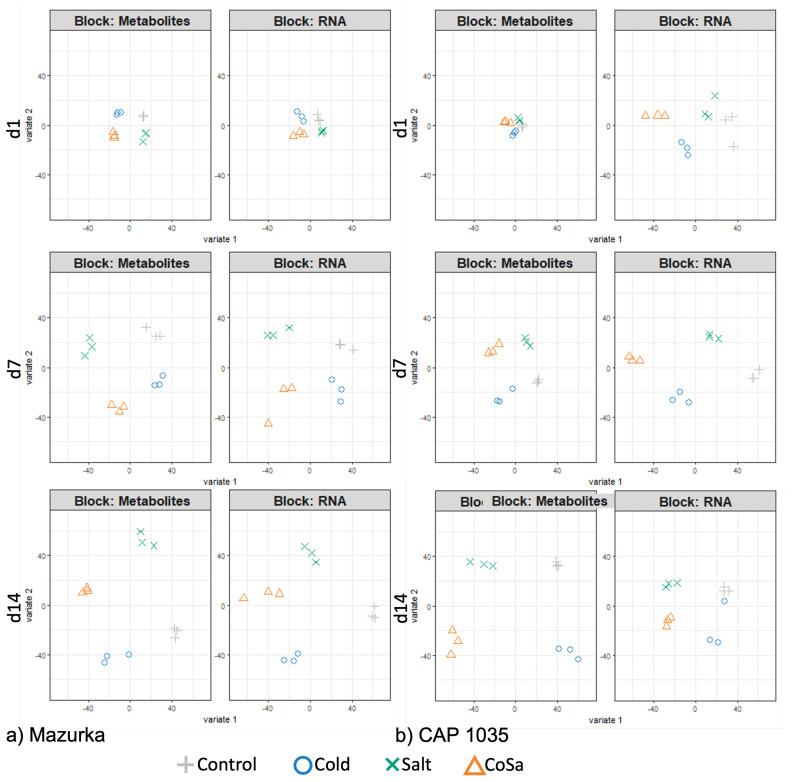
Scatter plot of multi-omics analysis (DIABLO) show the increasing response over time for Mazurka (**a**) and CAP 1035 (**b**). Mass features and RNA transcripts were analyzed for each species separately at each harvest time point. Each point represents one sample. Different colors and symbols represent different treatments: control = grey cross, cold = blue circle, salt = green x, CoSa = orange triangle.

**Figure A14:**
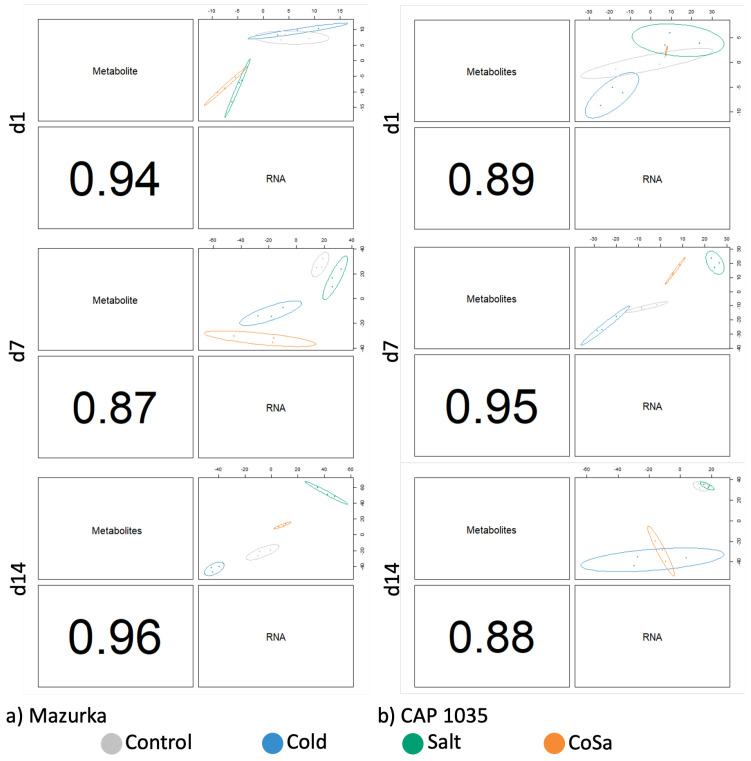
Overview DIABLO. A global overview of the correlation structure at the component level for each group according to the harvest time point (data for Mazurka is shown in (**a**) and for CAP 1035 in (**b**)). All components across the different data sets for a given dimension are plotted. Different colors represent different treatments: Control = grey, Cold = blue, Salt = green, CoSa = orange.

Sample plots of all six groups displayed a clear discrimination of each treatment samples with both the metabolome and the transcriptome data (see Figure 7a,b). In addition, different stresses were able to influence the metabolome and transcriptome profiles of both species after only one day. Samples of the combination treatment were separated from salt and cold samples after 1, 7 and 14 days, e.g., the early and the late response of the combination stress can be distinguished from each one of the stresses alone. The latent components in all six groups of both metabolome and transcriptome blocks were highly correlated, thus, DIABLO is able to model a good agreement between the data sets (see Figure A14a,b).

**Figure 8:**
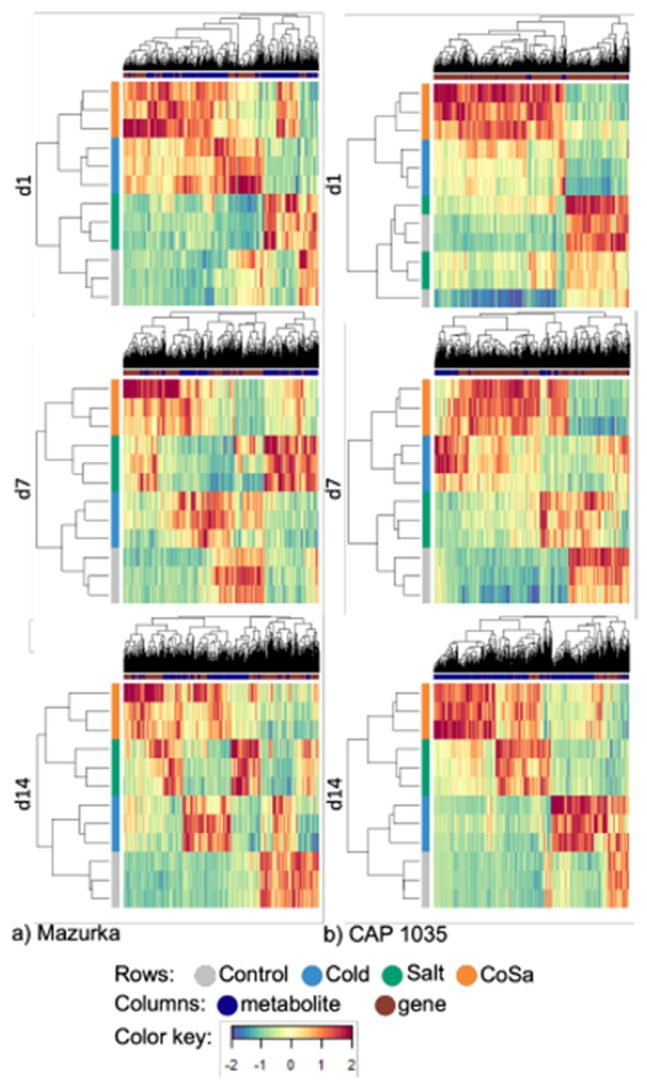
The hierarchical cluster of the multi-omics analysis (DIABLO) highlights the correlation between transcriptome, metabolome and stress treatment in Mazurka (**a**) and CAP 1035 (**b**). Clustered image map of the multi-omics dataset for all six groups according to harvest time point. Treatments are represented in rows and variables (RNA transcripts or mass features) in columns. Colors indicate the correlation between samples and variables where red means positive correlation, green to blue means negative correlation and yellow means no correlation.

The clustered image map performed with all DEG and mass features of all six groups showed a good classification of the four treatments (see Figure 8a,b). Interestingly, some of these genes and metabolites are only related to one treatment, e.g., low temperature or salinity or the combination. Other mass features are highly correlated to more than one treatment. Further, all treatments affected some mass features or genes after only one day in both species. Many genes and mass features are only affected by the combinatorial treatment and not by single chilling temperatures or salinity. Further, chilling temperature and salt treatments affected genes and mass features which were not altered by the combination treatment. This indicates a unique response for each abiotic stress in both species.

**Figure 9:**
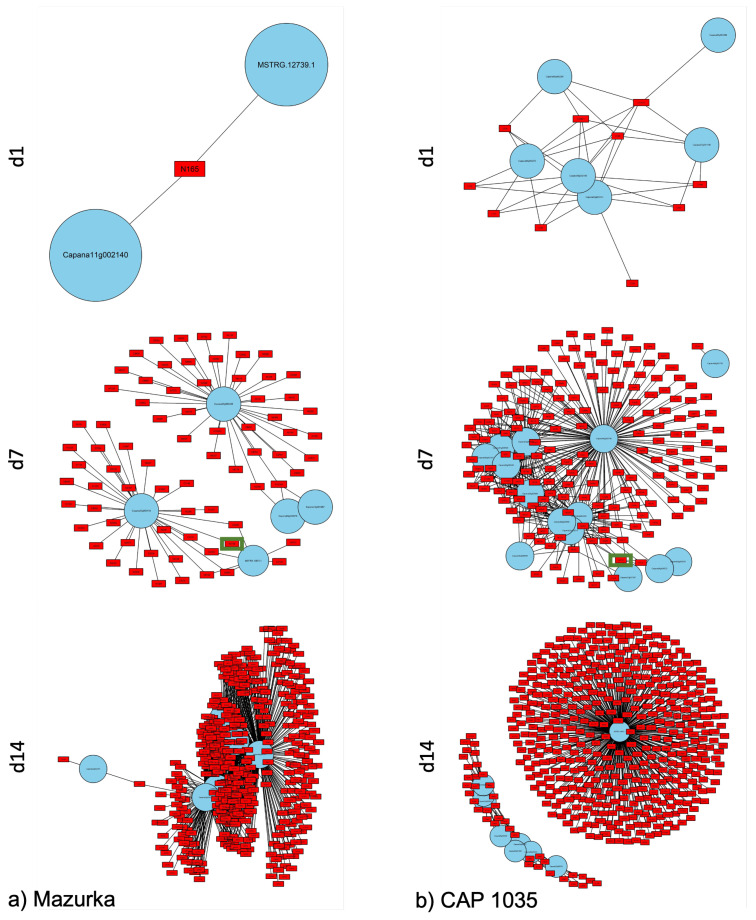
Network analysis of all secondary metabolites genesin Mazurka (**a**) and CAP 1035 (**b**). Secondary metabolite genes that correlated to at least one mass feature with r > 0.9 for our six groups were used for a network analysis. Genes are plotted with blue circles and mass features with red rectangles. At d7, putatively annotated metabolites are highlighted in green.

To identify networks related to secondary metabolism in *Capsicum*, a network analysis was performed using genes that were annotated to secondary metabolite pathways using the MapMan ontology [70] and all mass features that had a correlation coefficient with these genes of ≥0.9 (see Figure 9a,b, and supplementary Table S2 for the full list of involved metabolites). The number of mass features increased from day 1 to day 14 in both species.

In total, the network analysis identified 25 genes involved in secondary metabolite pathways. Those genes were further analyzed for their (putative) function. In our analysis, the identified genes were either induced or repressed for any stress condition at the respective harvest time point. According to the Mercator annotation [69,71], those genes were involved in different secondary metabolism pathways such as p-coumaroyl-CoA biosynthesis, flavonoid biosynthesis, carotenoid biosynthesis, terpene biosynthesis, and mevalonate pathway. In addition, we searched for orthologous or homologous genes with more than 85% query coverage and 90–100% sequence identity. For both species, all hits belong to the family of Solanaceae. In total, 18 genes out of the 25 have a putative annotation. For Mazurka, one gene was identified at d1, 5 at d7, and 6 at d14 (see Table 3). For CAP 1035, 6 genes were observed at d1, 14 at d7, and 9 at d14 (see Table 3). Only a set of 5 genes were detected in both species at the same harvest time point, and 5 genes are part of the networks at more than one time point of one species.

Mass features identified in the network analysis as highly correlated to secondary metabolism genes were annotated to known compounds according to their mass to charge ratio (*m*/*z*) and retention time (for a list of all mass features in the network analysis: see supplementary Table S2). Only nine mass features could be putatively annotated to known secondary metabolites (see Table 4). Further, according to their loading weights on each component within the respective group, they were also annotated to a specific stress treatment. In Mazurka at d14, all of the putatively annotated metabolites were annotated to the combination treatment while at d7 in Mazurka and CAP 1035 groups they were annotated to salt treatment (see Table 4).

This multi-omics analysis highlights the correlation between transcriptomic and metabolomic data. The performed analysis for the whole data sets in both species revealed a correlated increase for enriched genes and metabolites, especially for the combinatorial stress treatment. By reducing the transcriptomic data set to genes from the secondary metabolism, we were able to connect a few metabolites in both species with responding genes.

## 4. Discussion

Plants have to cope as sessile organisms with changing environmental conditions such as abiotic stresses. Often, biosynthesis of plant secondary metabolites (PSM) is stimulated [86,87,88,89] to overcome the resulting oxidative stress [8,90,91,92,93,94,95,96,97,98]. Consequently, abiotic stress treatment can influence the proportion and presence or absence of many PSM in all plant tissues [28,29,30,31].

Pepper (*Capsicum* sp.) is cultivated worldwide and a huge amount of biomass residual remains after harvest in horticulture. Different cultivated pepper species contain various PSM which have properties that make them useful for the pharmaceutical, agricultural and cosmetic industries [10,11,12,14,15]. Various studies investigated the effect of mild abiotic stresses under controlled growing conditions for tomato and pepper in green residuals, indicating a probable expansion for the value chain of horticultural fruit production, by extraction of induced PSM from remaining green residuals [18,20,52,99,100].

Therefore, we investigated in our study the effect of chilling temperature, salinity and a combination thereof on two different *Capsicum* species in the vegetative state focusing on a mid- to long-term response (harvest time points: 1, 7 and 14 days after stress induction). We specifically focused on metabolome and transcriptome profiles, and performed a multi-omics analysis to reveal the correlation between them. Several metabolites which correlated with the stress treatments were putatively annotated according to their spectral signature. By comparing the underlying response network of two species, we highlight differences in the investigated bell pepper Mazurka and the chili CAP 1035.

In general, we observed an increasing number of affected transcribed genes and mass features over the period of harvest in both species. Induction of pathways in response to abiotic stress results in activities of genes, which can result in mechanisms to overcome stress effects and also in accumulation of newly synthesized metabolites. Overall, the applied mild abiotic stresses resulted in few significant changes of the transcriptome. This is in accordance with a previous study based on phenotype data performed under equivalent conditions [52].

### 4.1. Transcriptional Mid- to Long-Term Response to Chilling Temperature and Salinity

According to the performed PCA with our transcriptome data set, the response differs only little between the three different stresses after one day and more differently the longer the stresses last (see Figure 2a,b), while control samples form a maintained cluster. In Mazurka, this was supported by the cluster analysis (see Figure A3) grouping all samples from d1 and some additional control samples from d7 in one cluster and all other sample in a second cluster.

**Figure A15:**
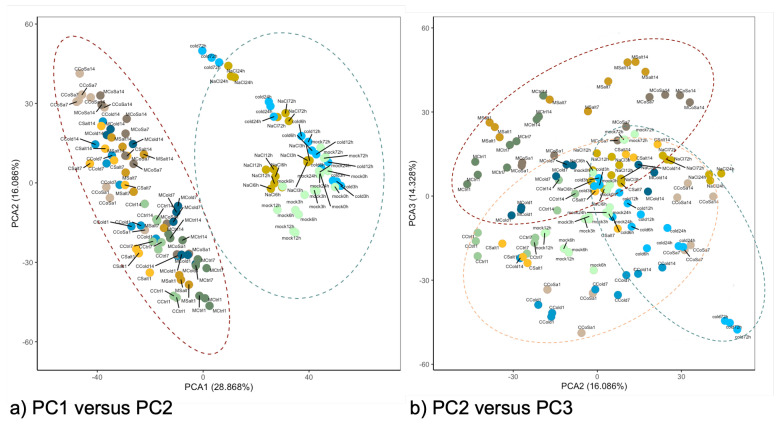
PCA from a combined analysis of Mazurka and CAP 1035 in comparison with published data from Kang et al. RNASeq reads were mapped to an artificial transcriptome comprimising transcriptomes coming from Mazurka (M) and CAP 1035 (C) and Kang et al. (lighter colors) [59] (see also methods). Shown in (**a**) is the principal component 1 (PC1) versus principal component 2 (PC2), indicating that the highest variation in the combined analysis is coming from the two different studies/species (red eclipse: our data, grey eclipse: Kang et al. data). In (**b**), the principal component 2 (PC2) is shown for principal component 3 (PC3), indicating that the next level of variation is influenced by the species (starting M versus starting C, red eclipse: Mazurka data, orange eclipse: CAP 1035 data, grey eclipse: Kang et al. data). Control samples (Ctrl) are indicated in green, cold samples (Cold) in blue, salt samples (Salt) in orange, and samples with a combination of salt and cold (CoSa) in brown.

In 2020, Kang et al. published a transcriptome analysis of sweet pepper subjected to heat, cold, drought and salinity [59]. They used plants of almost the same developmental stage and leaf samples as described in our study. The applied cold stress was at a constant 10 ℃ (day and night), and instead of 200 mM NaCl they applied 400 mM NaCl. Kang et al. investigated the early stress response between 3 h and 72 h after stress induction. PCA, performed with our data set and the published data, showed that the highest variance is determined by the studies and not the species (see Figure A15a).

The early response of the published data is characterized according to GO enrichment by a general oxidative stress and ROS production under any tested abiotic stress condition [59]. In addition, an enrichment for transcribed genes involved in a (hyper-)osmotic response and a general starvation response was observed under chilling temperatures. While under salt treatment, genes involved in a more general stress response including water deprivation were enriched.

**Figure 10:**
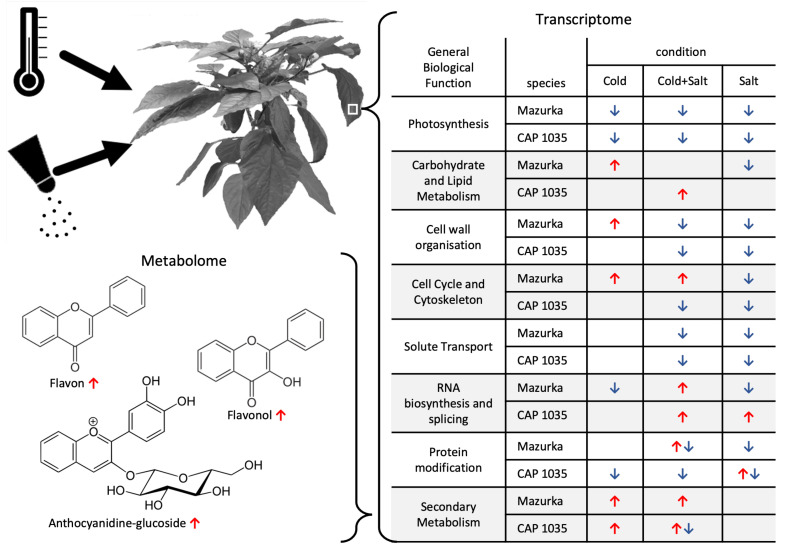
Mid- to long-term response to mild abiotic stress in *Capsicum* sp. Summary of the observed mid- to long-term response in leaves of *Capsicum* to chilling temperature, salt treatment and a combination thereof. A significant enrichment (red arrows) or depletion (blue arrows) in different biological pathways and for some putatively annotated secondary metabolites is highlighted.

The aforementioned enrichment of pathways of an early response were not observed in our data obtained for similar stresses in milder application. Instead, the overrepresentation analysis (ORA) showed an enrichment for up-regulated genes of the secondary metabolism in both species (see Figure 3 and Figure 4) for chilling temperatures and the combinatorial stress but not under salt stress treatment, and a more diverse pattern of biological functions for down-regulated genes at any time point (see also Figure 10).

In general, **chilling temperature** affects many processes such as photosynthesis, carbohydrate metabolism, polyamine synthesis, reactive oxygen species (ROS) scavenging, protein folding, stabilizing cell structure, cell membrane integrity, and chromatin remodelling [101,102,103,104,105,106].

In particular, cold stress will increase chlorogenic and phenolic production, where the latter is followed by incorporation into the cell wall as suberin or lignin [107,108,109]. In this respect, induction of secondary metabolism in **Mazurka** is following the results of previous publications. Additional for d14, we observed an enrichment of up-regulated ceramidase activity (part of sphingolipid metabolism), cell cycle control via cyclin B (CYCB), and general cell wall organisation. Ceramidase will disrupt ceramide (not detected in our metabolome profile due to solubility). After cold acclimation, a decrease in ceramide content was described for plasma membranes of *Arabidopsis thaliana* and oat [110,111]. These changes may contribute to hydration of the plasma membrane that could, in turn, increase plasma membrane stability during cold stress [112]. In fact, not only the plasma membrane will be affected. Chloroplasts are often influenced by cold treatment and sphingolipids are suggested to be structurally important in the envelope membrane of chloroplast for chilling tolerance [112,113]. CYCB is a member of the cyclins, and mediates G2-to-M-transition. In rice, the expression of the transcription factor OsMYB3R2 is stimulated under cold stress, which induces the expression of several G2/M-specific genes such as OsCYCB_1_. As a result, OsCYCB_1_ overexpression increased the resistance to cold stress [114]. Further investigation may highlight the role of CYCB in cold tolerance in *Capsicum sp*.

In addition, down-regulated genes involved in photosynthesis were enriched at d7 and d14 (see also Figure 10). CO_2_ assimilation of leaves and net photosynthesis rate will be reduced under chilling temperature [115,116], which was supported as well by our data. We also observed an enrichment of down-regulated pre-mRNA splicing pathways at d14. Calixto and colleagues recently reported the impact of chilling temperatures on alternative splicing in *Arabidopsis thaliana* [117]. They showed a dynamic process in the early response to chilling temperature starting as short as 30 min after reduction of temperature. As we find the down-regulation of pre-mRNA splicing pathways at d14 (late response), this may indicate a transition to a next state of either tolerance and/or adaptation and further experiments are necessary to elucidate the role of (alternative) splicing in the late (chilling) stress response.

A weaker effect of chilling temperature was observed for the transcriptome of the pungent pepper **CAP 1035** than for the cultivated Mazurka. Pathways of the secondary metabolism were induced (see discussion above), but we detected no influence on cell cycle, lipid metabolism or cell wall organization. Furthermore, pathways of the photosynthesis were down-regulated (see discussion above). In addition, CAP 1035 showed no effect on the pre-mRNA splicing under chilling conditions.

Instead, down-regulated genes of terpene biosynthesis and leucine-rich repeat-receptor kinases XII (LRR-XII) were enriched at d7. Terpene synthase genes influence photosynthesis, electron transport, developmental regulation and membrane architecture [118]. LRR are the largest subfamily of receptor kinases in plants [119,120], defining 14 subfamilies [120,121,122]. For most of the members, the biological function is still elusive, while the few members with a known biological function are involved in plant growth, developmental and physiological processes; hormone perception; brassinosteroid (BR) signaling; and defense responses to bacterial pathogens [123,124,125,126,127]. Whether down-regulation of photosynthesis pathways, terpene synthesis and inactivation of LRR-XII are connected in hot pepper should be confirmed in additional studies.

Interestingly, the reaction of *Capsicum* species in our present study to chilling temperature behave opposite to tomato species of a former publication [20]. In pepper the cultivated species Mazurka showed a strong induction of many different pathways (see above), while the effect in a cultivated tomato line *Solanum lycopersicum* was only mild: only a few genes were induced in tomato, among them UDP sulfoquinovose synthase and cyclin D. The opposite was observed for the pungent species and the wild *Solanum pennellii*. We found a weak effect for CAP 1035 (for discussion see above), while the response in the wild *Solanum pennellii* was strong and dominated by photosynthesis pathways and induced genes for perception of phytohormones [20].

For the **salt stress** in **Mazurka**, we found no enriched category for neither up- nor down-regulated after one day of treatment (see also Figure 3 and Figure 10). This is in accordance with data from Kang et al. [59], also showing a much lower number of influenced genes by salt than by chilling temperature. In addition, we detected no enrichment for up-regulated genes at d7 and d14.

It is known that salinity will induce oxidative stress in plants [25,128]. However, in leaf or root peroxisomes from salt-treated plants, no important changes in O_2_ or H_2_O_2_ concentration have been reported [129,130,131], partly due to increased antioxidant enzyme activity. Lack of up-regulated gene enrichment in our data could therefore be explained by changes in enzymatic activity to scavenge ROS, however this requires further investigation.

Instead, an enrichment of down-regulated genes involved in photosynthesis could be observed at d7 and d14. This is in accordance with previous publications [132,133], where reduced photosynthesis was associated with a decrease in stomatal conductance, transpiration rate, net photosynthesis rate and chlorophyll content under salt stress treatment.

In addition, genes involved in cell wall integrity were substantially down-regulated at d7 and d14. High salinity is a disadvantegous environmental stress, for whom the negative impact on growth and yield of crops is known. An accumulation of salt (especially sodium) in plants confers both ion toxicity and osmotic stress, which in turn affects the morphological, physiological, biochemical and metabolic status of a plant [134,135]. Reduction of cellulose content [136,137], disruption of the cross-linking of pectins [138], and accumulation of lignin biosynthesis [139] are common phenomenons due to high salinity. Our applied mild salt stress indicates the transcriptional changes for cellulose and pectin but not lignin.

Solute transport, protein modification, and cell cycle and microtubule integrity were also negatively affected at d7 and d14. Cell cycle integrity depends as well on microtubule organization, therefore, the observed enrichment of down-regulated genes involved in kinesin microtubule-associated motor protein activity may directly alter the cell cycle integrity. In addition, it is known that microtubule rearrangement and depolimerization is necessary to establish salt tolerance in *Arabidopsis thaliana* [140].

For **CAP 1035** under salinity treatment, we observed a similar pattern as described for Mazurka with two exceptions. First, we did not observe an enrichment of down-regulated genes involved in cell cycle at d7 and d14. This may probably highlight that reorganisation of microtubular networks in CAP 1035 are less dramatic than in the cultivated pepper Mazurka. Second, at d1, up-regulated genes were enriched, that are involved in the transcriptional regulation of RNA biosynthesis via WRKY transcription factor (WRKY TF) and protein modification via G-lectin protein kinase.

WRKY TF are known for their role in regulating the abiotic and biotic stress tolerance in plants [141,142]. During the last 15 years, a growing number of publications suggests that certain WRKY TFs regulate the production of valuable natural products by regulating metabolite biosynthetic genes including the secondary metabolism [143,144,145,146]. G-lectin protein kinases form the second largest subfamily of lectin-like receptor kinases, known to be involved in plant-microbe interaction [147]. In addition, L-lectin protein kinases are involved in salt stress tolerance by sensing and transduction of external Na^+^ [148], and activation of MPK3/MPK6 pathways leading to higher ethylene production and downstream ROS accumulation [149,150].

In this regard, our data highlights the early to mid-term response to salinity, albeit the direct connection of WRKY TF expression, protein modification via G-lectin protein kinase and reorganisation of the cell wall with salt stress response in *Capsicum annuum* will be subject of further studies.

### 4.2. The Transcriptomic Response to a Combination of Abiotic Stresses Differ from Single Stress Responses in Cultivated Bell Pepper

Plants growing in the field have to cope with multiple different stresses. Those stresses do not necessarily act additive, but can also provoke antagonistic effects [20,151,152,153]. Our transcriptomic data support these findings (see Figure 3, Figure 4 and Figure 10).

For **up-regulated genes in Mazurka**, we observed an induction of secondary metabolism under the combinatorial stress, but we did not find an enrichment of ceramidase activity, CYCB-mediated cell cycle control and cell wall organization. For those pathways, chilling temperatures and salinity seem to have antagonistic effects. Instead, clade A phosphatases were up-regulated. Protein phosphatase 2C (PP2C) is known to be involved in abiotic stress responses in plants, such as salt tolerance, drought response, osmotic stress or ABA signaling [154,155,156,157]. It can be speculated that mild chilling stress acts additive to salinity and caused the observed enrichment of those phosphatases.

In case of **down-regulated genes in Mazurka**, the transcriptional response covers greatly that for salinity or chilling temperature as single stresses, with the exception that no enrichment of down-regulated cell cycle and cytoskeleton organisation were observed. We hypothesize that the chilling temperature may have slowed the responding processes for salinity, as discussed previously [35,158,159], thereby masking the inhibition observed under single stress treatment.

Like for Mazurka, we observed an enrichment of secondary metabolism in **CAP 1035**, whereas we did not detect the enrichment of **up-regulated** G-lectin protein kinases under the combinatorial stress treatment. Here, the additional chilling temperature stress may have declined the signaling pathways for G-lectin protein kinases.

The transcriptomic response of **down-regulated genes** in CAP 1035 resemble the responses to single stresses with two exceptions. First, inhibition of terpene biosynthesis (chilling stress) and kinesin activity (salinity) were not observed. Second, preinitiation of DNA replication was inhibited instead. In maize, a delay in S-phase and inhibition of G2-to-M phase was published for salt stress treatment [160,161], while an increased cell cycle time was observed in the meristem of leaves exposed to chilling temperature [162]. Here, further studies may elucidate the impact of salt stress to suppress the activation of the cell cycle by chilling temperature in *Capsicum* sp.

Overall, the high similarities of responses (to our applied abiotic stresses) between the cultivated pepper Mazurka and its relative CAP 1035 may reflect a lower grade of domestication for pepper than for other species, e.g., strawberry or tomato [20,163,164].

### 4.3. Metabolic Responses Increases from Mid-to Long-Term Response

Chilling temperature, salinity and their combination affected the metabolome profiles of both species (see Figure 6 and Figure 7). First, all mass features were used to investigate the effect of stresses on the metabolome profiles in each species.

After one day, no separation between treated and untreated samples was observed. However, the effects of different stresses were first detected after 7 days and further strengthened at day 14. In addition to separation of samples from different time points, samples from the different stress treatments formed distinct groups at day 14 (see Figure 6). The same pattern was detected after analyzing samples from each time point separately using only mass features that are correlated to the stress by DIABLO (see Figure 7). In both, Mazurka and CAP 1035 the discrimination between samples of each stress increased over time. In addition, the number of mass features that are induced or repressed significantly between treated and untreated samples increased from day 1 to day 14 (see Table 1). Therefore, we conclude that the effect of abiotic stress on the metabolome profile also increased during time. This result agrees with an earlier report using the same species and cultivation conditions where the content of flavonoids was increased after exposing different *Capsicum* sp. to chilling temperature, salinity and a combination thereof from seven days on, and these treatments also inhibited plant growth and reduced the leaf chlorophyll fluorescence index mainly after 14 days [52].

Moreover, different studies proved the effect of abiotic stress on metabolites in plants [52,99,100,165,166]. Graveobioside A and cynaroside concentrations in bell pepper leaves were enhanced after exposition to UV light and salt stress, and the highest concentrations were reached by combining both treatments [100]. These metabolites were also induced by cold temperatures, salt treatments and the combination of cold and salt treatments, whereas apiin and apigetrin showed only a mild response to these stress treatments [52]. In addition, in vitro concentration of solasodine was increased in the *Solanum nigrum* callus growing under salt stress [165].

However, abiotic stress can also decrease the amount of secondary metabolites in plants. For example, ascorbic acid concentration and total phenolic content in *C. annuum* “Chili-AS Rot” were decreased under drought stress while capsaicin and dihydrocapsaicin concentrations increased in stressed plants [166].

The clustered image map of our six groups showed correlation of differentially enriched genes and mass features under different stress treatments (see Figure 8). For chilling temperature and salinity single stresses, positive or negative correlation between various genes and mass features were revealed and some of these correlations were also observed for the combined treatment. However, many genes and mass features are only correlated to one of the three different treatments. We conclude that each stress treatment (single and combinatorial stresses) releases a unique mark in the metabolome and transcriptome profile.

Different abiotic stresses can interact, which will cause a unique response different from the single stress [34,35,167]. For example, the osmoprotectants glycine betaine and trehalose were induced in *S. lycopersicum* plants treated with combined heat and salinity stress while proline was induced in plants challenged with salinity stress only [167]. In addition, the unique response can be affected by different factors like the species, the age of the plant and the degree of the stresses [34]. For both species, our results for the metabolome profile reveal that the response of Mazurka and CAP 1035 to the combination of chilling temperature and salinity is different from their response to the single stresses.

Besides the increasing amount of affected mass features (see Table 2) in both cultivars over all harvest time points, only for CAP 1035 we also observed a drop down in the absolute number of affected genes at d14 under chilling temperatures to almost the level of d1 (see Figure 2). In general, this may indicate an early acclimation process visible in the pungent pepper CAP 1035 and is in accordance with a former publication [168]. Ref. Ou et al. [168] showed an increase of soluble sugars and some amino acids mediated by chilling temperature and low irradiance over a period of 15 days in various pepper species, among them *C. annuum* and *C. chinense*. They also checked the transcriptional level of *C-repeat binding factor 3* and cold-regulated genes, which showed the highest transcriptional levels at days 5 (*C-repeat binding factor 3*) to 15 (cold-regulated genes) followed by decreasing levels until day 25 (end of the investigated time line). Looking at the ORA analysis in our study (see Figure 4), this acclimation seems to involve for example the repression of photosynthesis genes at day 14, however not the genes in the secondary metabolism, which correlates with the ongoing increase of metabolites also in CAP 1035.

Increased amounts of mass features at d14 suggest increased protein activity despite reduced amounts of differentially expressed genes in CAP 1035. The level of transcription determines protein abundance, however protein activity is also modulated by post-translational modifications. Post-translational modifications can influence protein function, half-life and interactions to alleviate the potential damage of environmental stress [103,169] and cold temperatures in particular [170].

In addition, our transcriptomic data of early harvest time points (d1 and d7) indicated a slightly higher amount of differentially enriched genes in the pungent pepper CAP 1035 than in Mazurka (see Figure 2). This finding, in the context of a putative acclimation afterwards in CAP 1035, is supported by a recent publication of Zhang et al. [171]. The authors investigated the early response of a cold sensitive and a cold resistant cultivar in *C. annuum*. Thereby, they showed a similar pattern with slightly more differentially enriched genes under cold stress in the cold-resistant cultivar than in the cold-sensitive cultivar. Further studies may investigate the underlying biological processes for a more detailed insight of acclimation and tolerance to chilling and cold temperatures in pepper.

### 4.4. Multi-Omics Network Uncover Gene-Metabolite Relations in Secondary Metabolite Pathways

We further focused on identifying gene-metabolite networks related specifically to secondary metabolism. Genes annotated to secondary metabolite pathways (according to the MapMan ontology) were used to perform a detailed network analysis and the cut off was set to ≥0.9 (see Figure 9). In both species, the number of genes and mass features increased during time (for genes see Table 3). This indicates that more secondary metabolite pathways were influenced as a response to abiotic stress in both species. Some of these mass features were putatively annotated to known metabolites (see Table 4).

For the CAP 1035 **network at d1**, our analysis mainly revealed fundamental structural genes of the secondary metabolism, such as phenylalanine ammonia-lyase and trans-cinnamate 4-monooxygenase (see Table 3), involved in the first processing steps of the phenylpropanoid pathway. In contrast, the network of Mazurka showed a putative WPP domain-interacting tail-anchored protein 2 (WIT2). WIT2 is known to play a pivotal role in nuclear shape determination and nuclear envelope docking of RANGAP proteins in root tips [172,173]. WIT2 was included in the network analysis based on its MapMan annotation and the influence of WIT2 on secondary metabolite pathway remains elusive.

The **network analysis of d7** showed 5 genes for Mazurka. Two of them (Capana03g000578: 4-coumarate_coenzyme A ligase and Capana00g003499: phenylalanine ammonia-lyase) belong as well to the fundamental structural genes involved in early steps of the flavonoid biosynthesis pathway, while Capana12g001287 is annotated to later steps in this pathway and Capana05g000154 and MSTRG.13875.1 to the mevalonate pathway (part of terpene biosynthesis). For CAP 1035, we observed a similar pattern with 7 of 14 genes belonging to the terpene biosynthesis and the remaining genes involved in flavonoid biosynthesis (5 structural genes) or the early processing steps in phenylpropanoid pathways (2 genes).

The induction of terpene biosynthesis, in particular via the mevalonate pathway, is a known response in plants to cold and salinity [20,116,174,175,176], that is supported as well by our results. In addition to general terpene biosynthesis, low-temperature regimes may induce carotenoid accumulation [177]. In particular for CAP 1035 we identified three genes (putatively) involved in carotenoid biosynthesis and their specific function in response to chilling temperature and salinity in *Capsicum* sp. might be the subject of further studies.

Many of the putatively annotated compounds at d7 are flavonoids, which form a major group of secondary metabolites and contain up to 10,000 different compounds across the plant kingdom, subdivided into different groups [16,178,179]. Flavonoids exhibit a wide range of biological activities *in planta*, including the control of plant development, plant–microbe interactions and plant–animal interactions [178,180] and many flavonoids were detected in different tissues of *Capsicum* sp. including leaves [4,181].

In addition, flavonoids, among other secondary metabolites in plants, are induced as a response to abiotic stresses, like chilling temperature and salinity, to reduce the oxidative stress caused by the accumulation of reactive oxygen species (ROS) in cells and, thus, avoiding cell death [28,29,30,31,182]. Flavonoids also have beneficial health properties against a number of chronic diseases when taken up in the diet (reviewed in Knekt et al. [183], Tohge and Fernie [184]), which makes them of interest for food and pharmaceutical industries.

The flavonoid **apigetrin** was putatively annotated in the salt stress network of Mazurka at d7. Apigetrin has many cellular bioactivities, such as regulation of oxidative stress, and induction of apoptosis [185]. Apigetrin was also previously detected in *Capsicum* sp. [31,100,186] and induced in bell pepper leaves as a response to the combination of cold and salt stress [52].

In addition, **kaempferol, luteolin, quercetin** and many of their glycosides were determined in *Capsicum* sp. [52,74,77,81,82,83,100]. Two luteolin glucosides (cynaroside and graveobioside A) were detected in the leaves of Mazurka and CAP 1035 and have already been shown to be accumulated by chilling temperature and a combination of chilling temperature and salinity [52]. Due to their catechol structure, both may have an impact as an antioxidant acting as an efficient free radical scavenger [187]. Further, salt stress induced the content of kaempferol and quercetin in *Apocynum venetum* L. seedings [188]. In addition, a recent publication showed that kaempferol and quercetin will reduce the effect of salt stress in combination with warm temperature by their radical scavenging nature [189]. As a conclusion, chilling temperature, salinity, and their combination induced the amount of different valuable bioactive compounds in Mazurka and CAP 1035 leaves.

Again in both species, many fundamental structural genes of early steps in the phenylpropanoid pathway are present in the **network analysis of d14**. This is in accordance with the observed accumulation of p-coumaroyl quinic acid at d14 in Mazurka in samples of a combinatorial stress of chilling temperature and salinity.

Additional, we identified structural genes of terpene and flavonoid biosynthesis (as discussed above). **Rutin** is one of the putatively annotated compounds among others at d14 (see Table 4). It is considered to play a pivotal role in general plant protection mediated by environmental changes [190], most likely due to the radical scavenging capacities. It was also detected in different plants including leaves of *Capsicum* sp. [84,100,186]. Different studies correlated the accumulation of rutin to abiotic stress; for example, exposing tomato young leaves to nitrogen and general mineral limitation, chilling temperature, warmer temperature or elevated light led to the accumulation of rutin [20,99]. Further, UV-B radiation, cold and desiccation stress exposed to *Fagopyrum tataricum* leaves also caused an increase in rutin concentration [191].

The putatively annotated **cyanidin 3,7-di-O-beta-D-glucoside** is an anthocyanidine. Anthocyanins are PSM responsible for the color (e.g. blue, purple or red) of many plants, and split off from the flavonoid biosynthesis pathway. Cyanidin-3-O-glucoside is known to have a protective effect on DNA cleavage, a dose-dependent free radical scavenging activity and significant inhibition of xanthine oxidase activity [192]. It is investigated as pharmacological substance used in heart protection, cancer therapy and further medical applications [177,193,194,195]. The accumulation of anthocyanins due to cold and chilling temperature is well known and supposed to be involved in cold acclimation and tolerance [196,197,198,199], and is also supported by our results.

Overall, even mild forms of chilling temperature, salinity and their combination influenced the metabolome and the transcriptome profiles of both Mazurka and CAP 1035. The influence of stress increased during the time in both species. In addition, each of the three treatments stimulate a unique response for each species. Further, chilling temperature and salinity interact and impact differently in both species. Finally, some bioactive compounds were annotated to known secondary metabolites of industrial interest; and correlated with known genes of the secondary metabolism.

## 5. Conclusions

Our study is the first detailed transcriptomic and metabolomic analysis of mid- to long-term response to various mild abiotic stresses and a combination thereof in leaves for a sweet pepper variety (*Mazurka*) and its hot relative (*CAP 1035*). We focused on mid- to long-term time points to get some insights on the underlying molecular mechanisms. Nevertheless, our analysis is only a snapshot of three time points and one specific organ, which is rarely investigated. Further studies will be necessary to investigate and differentiate mid- and long-term response in further different pepper organs (e.g., root, shoot, different ages of leaves, flower, *…*).

We were able to show substantial alterations on the molecular level based on our transcriptome and metabolome analysis as a response to the stress treatment at three harvest time points but also between the species. This study shows that leaf transcriptome and metabolite levels are under great environmental influence, as only mild abiotic stresses result in stress-specific transcriptomic and strong metabolic changes with a substantial induction of compounds of industrial interest. Our study indicates leaves of bell pepper and chili under mild abiotic stresses as a valuable source for antioxidants such as flavonoids, as shown previously for tomato [18,19,20] and proposed for bell pepper [52,100].

Therefore, exposing the biomass residual of pepper after harvest to (mild) chilling temperature and salinity can increase the amount of the valuable metabolites and, thus, optimize the utilization of crops.

## Figures and Tables

**Table 1:** Final group details for each DIABLO analysis. Transcriptome and metabolome data were analyzed to define highly correlated genes (differentially enriched genes, DEG) and mass features for each species at each single harvest time point performing first a WGCNA followed by an ANOVA.

Species	Harvest Time Point	No. of Sig. DEG	No. of Sig. Mass Features
	Day 1	437	352
Mazurka	Day 7	2320	2040
	Day 14	5623	3385
	Day 1	1489	70
CAP 1035	Day 7	3413	956
	Day 14	1818	4691

**Table 2:** Increasing numbers of (unknown) mass features affected by stress treatments over the harvest period in Mazurka and CAP 1035. Spectra of all samples were analyzed regarding *m*/*z*, retention time and peak area. Compounds detected in stressed samples were compared with the respective control sample.

			Condition					
	Timepoint	Total No. of	Cold		Cold+Salt		Salt	
		Affect. Mass Features	Increase	Decrease	Increase	Decrease	Increase	Decrease
	Day 1	352	256	93	279	71	264	83
M	Day 7	2040	1314	686	1587	439	1530	500
	Day 14	3385	2262	1099	2652	726	2536	789
	Day 1	70	46	23	54	15	41	28
C	Day 7	956	766	159	754	194	559	366
	Day 14	4691	3690	729	3933	741	3578	1041

**Table 3:** (Putative) Annotation of genes detected by the network analysis of all six investigated groups. Sequences of the detected genes from the secondary metabolism were used to perform a BLAST analysis. A putative annotation is indicated in the column ‘Put.’. The induction (up) or repression (down) of the gene at the respective harvest time point is indicated in column ‘Reg.’. Harvest time points are indicated as d1, d7 and d14. *Capsicum* species are specified as C—chili CAP 1035, and M—cultivated pepper Mazurka.

Gene ID	Gene Name	Put.	Reg.	d1	d7	d14
Capana06g000272	trans-cinnamate 4-monooxygenase		up	C	C	C
Capana09g002190	phenylalanine ammonia-lyase-like		up	C		C
Capana09g002200	phenylalanine ammonia-lyase-like		up	C		C
Capana06g000273	trans-cinnamate 4-monooxygenase-like		up	C		C, M
Capana00g003499	phenylalanine ammonia-lyase	x	up	C	M	
Capana07g001146	acetyl-CoA acetyltransferase	x	up	C		
Capana11g002140	WPP domain-interacting tail-anchored	x	up	M		
	protein 2					
Capana06g001083	4-coumarate-CoA ligase 2	x	up			C
MSTRG.44643.1	chalcone synthase J-like	x	up			C
Capana02g002404	3-hydroxy-3-methylglutaryl-coenzyme A reductase	x	up		C	M
	reductase					
Capana05g002274	chalcone synthase 2		up			M
Capana01g000948	carotenoid cleavage dioxygenase 4	x	down		C	C, M
Capana05g002107	chalcone-flavonone isomerase 3	x	up		C	C, M
Capana03g000578	4-coumarate_coenzyme A ligase (4CL)		up		C, M	C, M
Capana08g001733	2-C-methyl-D-erythritol 4-phosphate	x	down		C	
	cytidylyltransferase					
Capana09g000404	beta-amyrin synthase-like	x	up		C	
Capana04g002519	bifunctional 15-cis-phytoene synthase		up		C	
Capana00g002736	chalcone-flavonone isomerase 3	x	up		C	
Capana06g002959	cytochrome P450	x	down		C	
Capana03g000892	flavonoid 3’-monooxygenase	x	up		C	
Capana06g000463	licodione synthase-like	x	up		C	
Capana02g000638	select squalene epoxidase 3-like	x	up		C	
Capana05g000154	acetyl-CoA acetyltransferase, cytosolic 2	x	up		M	
MSTRG.13875.1	hydroxymethylglutaryl-CoA synthase-like	x	up		M	
Capana12g001287	acetyl-CoA carboxylase 1	x	up		C, M	

**Table 4:** Putatively annotated metabolites from the network analysis of all six investigated groups. All annotated metabolites were enriched due to stress treatment and detected in positive (starting with C) or negative mode (starting with N). Harvest time points are indicated as d1, d7 and d14. *Capsicum* species are specified as C—chili CAP 1035, and M—cultivated pepper Mazurka.

Group	Compound	Stress	Putatively Annotated Metabolites	Reference
M d7	N2109	Salt	Isovitexin	[74,75]
			Vitexin	[75]
			Apigetrin	[52]
			Afzelin	[76]
C d7	N2744	Salt	Kaempferol-3-O-rutinoside	[74]
			Luteolin 7-beta-neohesperidoside	[77]
M d14	C3609	CoSa	p-Coumaroyl quinic acid	[78]
	N6446	CoSa	Sophoraflavonoloside	[79]
	N3794	CoSa	Myricitrin	[80]
	N6025	CoSa	Kaempferol	[81]
			Luteolin	[81]
	N3036, N3037, N4704	CoSa	Apiin	[52]
			Quercetin 3-O-rhamnoside 7-O-glucoside	[82,83]
			Rutin	[84]
			Cyanidin 3,7-di-O-beta-D-glucoside	[85]

**Figure A12:**
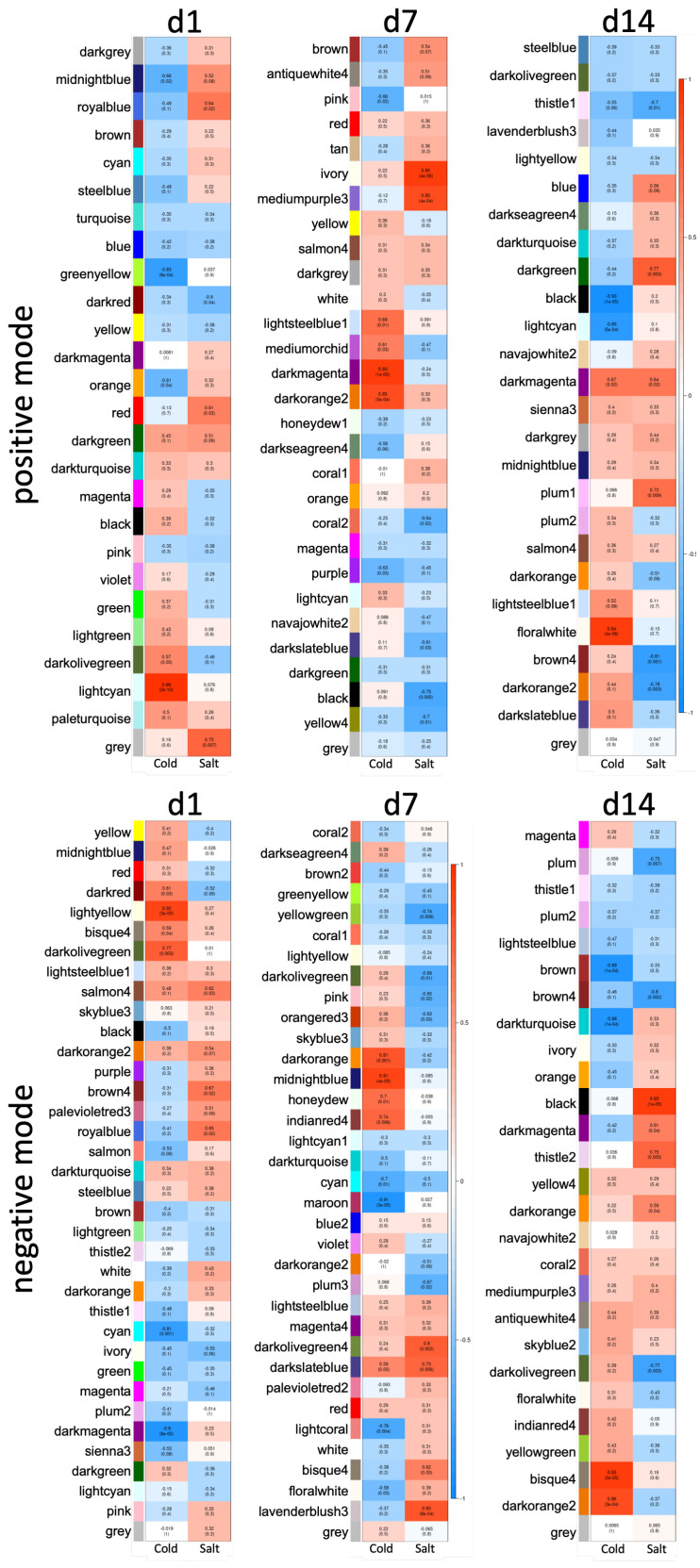
Module-trait correlation for the metabolome of Mazurka. Metabolic data (positive and negative mode) was used to perform a WGCNA. Shown here is the relationship for each identified cluster with the single stress chilling temperature (Cold), or salt (Salt) as indicated at d1, d7, or d14. On the y-axis, all identified modules are listed with their respective names. Color and number indicate the correlation between module and binary trait for eigengenes and numbers in brackets the *p*-Value of significance.

**Figure A13:**
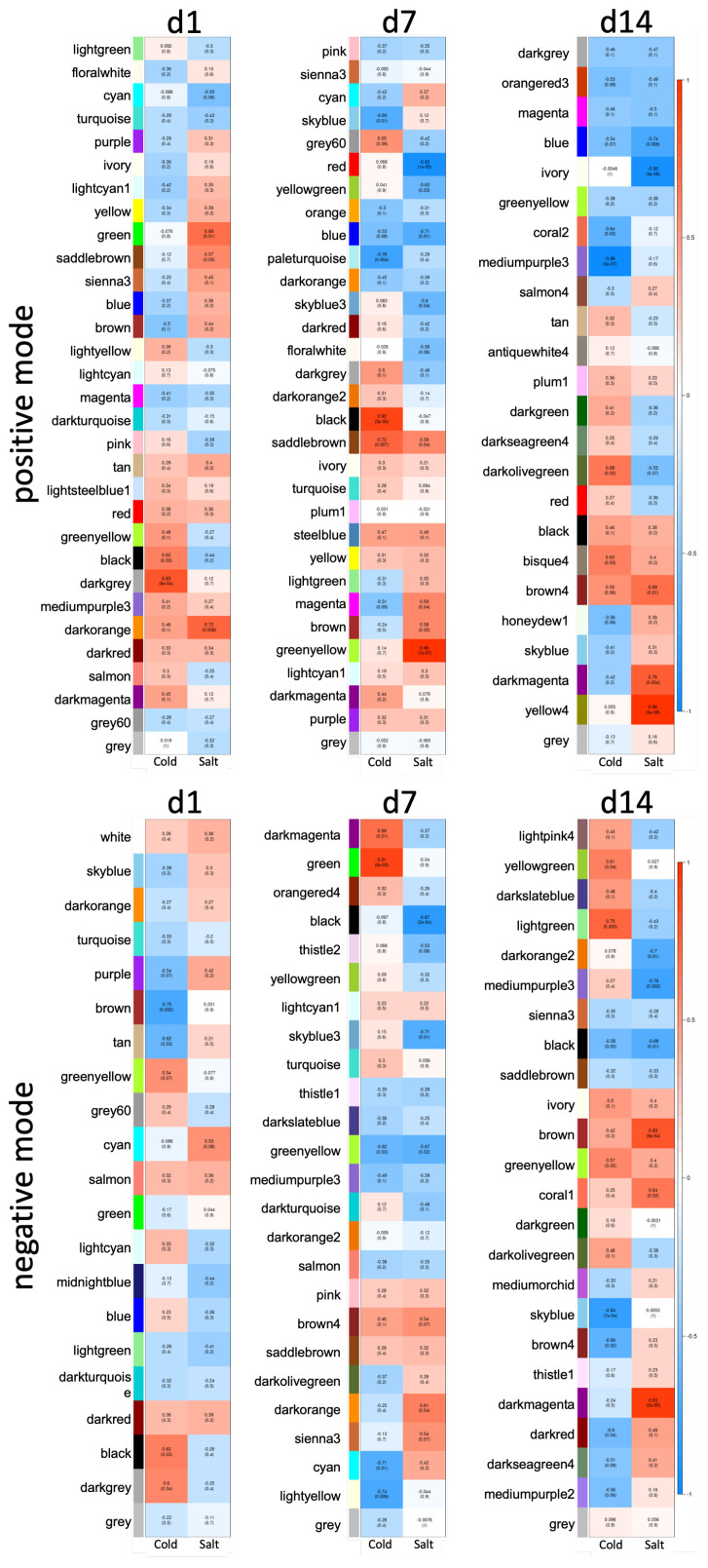
Module-trait correlation for the metabolome of CAP 1035. Metabolic data (positive and negative mode) was used to perform a WGCNA. Shown here is the relationship for each identified cluster with the single stress chilling temperature (Cold), or salt (Salt) as indicated at d1, d7 or d14. On the y-axis, all identified modules are listed with their respective names. Color and number indicate the correlation between module and binary trait for eigengenes and numbers in brackets the *p*-Value of significance.

## Data Availability

Publicly available datasets were analyzed in this study. This data is deposited in the NCBI database under the BioProject PRJNA641558: https://www.ncbi.nlm.nih.gov/bioproject/641558, accessed on 6 March 2022.

## Supplementary Materials

The following supporting information can be downloaded at: https://www.mdpi.com/article/10.3390/antiox11040671/s1: Table S1: cpm and toptags of the sequencing data for both species, Table S2: Overview experimental set up, oligo nucleotide list, and metabolite data for negative and positive mode.
